# Leveraging Microphysiological Systems to Facilitate Neutrophil‐Based Cancer Immunotherapy

**DOI:** 10.1002/advs.77572

**Published:** 2026-09-13

**Authors:** Shuai Shao, Markus Isfeld Diehl, Caroline N. Jones

**Affiliations:** ^1^ Department of Bioengineering The University of Texas at Dallas Richardson Texas USA; ^2^ Department of Biomedical Engineering UT Southwestern Medical Center Dallas Texas USA; ^3^ Program in Immunology Stanford University Stanford California USA; ^4^ Department of Otolaryngology‐Head and Neck Surgery (OHNS) Stanford University Stanford California USA

**Keywords:** cancer, immunotherapy, microphysiological system, neutrophil, organ‐on‐a‐chip

## Abstract

Neutrophils are the most abundant immune cells in human blood and serve as the first line of defense against infection. However, the role of neutrophils in cancer progression is complex and context‐dependent, and they are underexplored as therapeutic targets for cancer compared with other immune cell types. Microphysiological systems, also known as complex in vitro models, have emerged as powerful tools for deciphering immune cell‐cancer interactions and preclinical drug testing due to the advantages of real‐time visualization of cell behaviors, precise control of microenvironments, and incorporation of human cells over in vivo animal models. This review first summarizes the multifaceted roles of neutrophils in cancer as defined by in vivo animal models and highlights recently developed strategies to mobilize neutrophils for cancer treatment. Next, current applications of different types of microphysiological systems, including 3D spheroids, organoids, and microfluidic organ‐on‐a‐chip platforms, for investigating the role of human neutrophils in cancer and evaluating neutrophil‐based cancer immunotherapies are surveyed. Lastly, future opportunities for microphysiological systems to address key challenges in the clinical translation of neutrophil‐based cancer immunotherapy are discussed.

## Graphical abstract

Microphysiological systems are emerging as powerful human‐relevant platforms for studying neutrophil biology in cancer. Current applications of spheroids, organoids, and organ‐on‐a‐chip models are reviewed, together with future opportunities in behaviorome profiling, multi‐omics integration, personalized medicine, immune crosstalk, and multi‐organ systems to advance neutrophil‐based cancer immunotherapy.

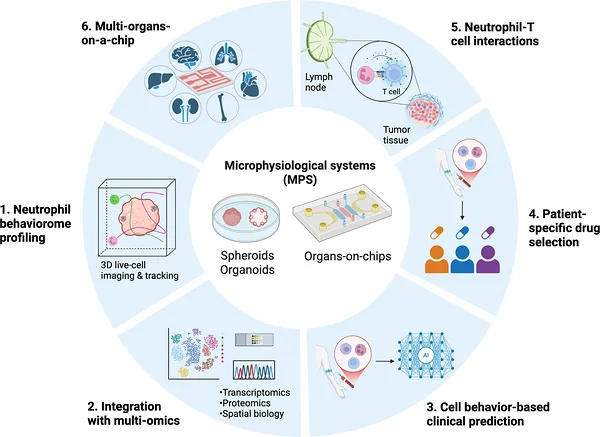

## 1 Introduction

The immune system comprises two major arms: the innate immune response and the adaptive immune response. Neutrophils are the key component of the innate immune response and are the most abundant immune cell type in humans, constituting around 50%–70% of all immune cells circulating in human blood [1]. Neutrophils are produced in the bone marrow and released into the bloodstream on the order of ∼10^11^ cells per day in adult humans under homeostatic conditions [2]. Neutrophils are terminally differentiated cells and, compared to other immune cell types, are considered short‐lived, with a life span ranging from 8 h to 5.4 days [1, 3, 4]. Neutrophils are known as the first line of defense against infections from pathogens such as bacteria and fungi. Once sensing microbial or inflammatory signals, neutrophils are rapidly recruited from circulation into sites of infection or inflammation through a tightly regulated cascade of events including adhesion to the endothelium and extravasation into tissues. Once recruited, these immune cells exert a broad range of effector functions, including migration, phagocytosis, degranulation, production of reactive oxygen species (ROS), and release of neutrophil extracellular traps (NETs), to eliminate pathogens and modulate inflammatory responses [3, 5].

Beyond host defense, neutrophils also play important roles in tissue homeostasis, including wound healing and tissue repair [6]. Cancer has been described as a “wound that does not heal” [7], and, accordingly, tumors can sustain persistent recruitment of neutrophils, contributing to chronic inflammation within the tumor microenvironment [6]. Analyses of human tumor specimens have identified neutrophils as one of the most abundant immune cell populations present in multiple cancer types [1]. Notably, this sustained infiltration is often associated with poor clinical outcomes, and neutrophils have been widely implicated in tumor‐promoting processes such as angiogenesis, immunosuppression, and metastasis [8]. At the same time, accumulating evidence also supports anti‐tumor functions of neutrophils under specific conditions, highlighting their functional programmability [1, 9]. Together, these findings position neutrophils as a double‐edged sword in cancer and underscore their complex and still controversial roles in tumor progression [1, 10].

Much of our current understanding of neutrophil biology in cancer has been derived from in vivo animal models, which have been essential for elucidating mechanisms of neutrophil recruitment, polarization, and function [11, 12]. These studies have also inspired the development of therapeutic strategies aimed at either inhibiting pro‐tumor neutrophil activity or amplifying their cytotoxic potential for cancer treatment [9]. However, only a limited number of neutrophil‐targeting drugs for cancer are currently undergoing clinical trials [1, 13], and unlike T cells and NK cells, no adoptive cell therapy based on neutrophils has entered clinical trials yet [5]. Translation of neutrophil‐based immunotherapies from mice to humans remains highly challenging due to fundamental differences between murine and human immune systems [12, 14]. Moreover, traditional in vitro and in vivo models have limited capacity to capture neutrophil‐cancer interactions in detail within the complex tumor microenvironment or to accurately predict human responses to therapeutics.

Microphysiological systems (MPS), also referred to as complex in vitro models, have emerged as powerful tools to address these limitations [15]. These bioengineered platforms, including spheroids and organoids as well as organ‐on‐a‐chip systems, can recapitulate key features of tumor biology using human cells [16], while allowing real‐time visualization and quantification of cell behaviors with the help of live‐cell imaging [17, 18]. In particular, organ‐on‐a‐chip models use microfluidic devices with defined compartments to spatially organize different cell types, and can incorporate dynamic fluid flow to mimic physiological processes [19, 20]. With precise control of biochemical and mechanical cues [21, 22], these systems serve as human‐relevant platforms to investigate the role of neutrophils in cancer and evaluate immunotherapies with high spatiotemporal resolution in well‐defined microenvironments. Hence, MPS can offer insights into neutrophil‐cancer interactions that are difficult to obtain using conventional approaches while maintaining a balance between complexity and scalability.

In this review, we first outline the diverse roles of neutrophils in cancer as established through in vivo studies and highlight emerging strategies aimed at harnessing or modulating neutrophil functions for cancer therapy. Next, we introduce the basics and benefits of MPS, including spheroids, organoids, and organ‐on‐a‐chip models for cancer immunology research. We then examine recent progress in the use of MPS to study human neutrophil‐tumor interactions and to assess neutrophil‐targeting therapeutic strategies for cancer. Lastly, we explore future directions through which MPS can advance the clinical translation of neutrophil‐based cancer immunotherapy, including neutrophil behavioral profiling, integration with multi‐omics technologies, personalized medicine, and engineering more complex, multi‐cellular and multi‐tissue systems.

## 2 The Role of Neutrophils in Cancer

In this section, we discuss both pro‐tumor and anti‐tumor mechanisms of neutrophils identified mostly in preclinical in vivo studies, followed by an overview of recent sequencing‐based research highlighting the heterogeneous states of neutrophils in cancer.

### 2.1 Pro‐Tumor Functions of Neutrophils

The majority of studies to date point to a tumor‐promoting nature of neutrophils [1, 23]. Elevated neutrophil counts in peripheral blood and an increased neutrophil‐to‐lymphocyte ratio have been correlated with poor clinical outcomes in human patients across a broad range of solid tumors [8, 11]. Other studies indicate that the presence of neutrophils within tumor tissue—commonly termed tumor‐associated neutrophils (TANs)—also correlates with a poor prognosis. Notably, a comprehensive meta‐analysis published by the CIBERSORT group in 2015 used gene expression data collected from ∼18 000 human tumor specimens across 30 cancer types and showed that neutrophils were associated with the worst survival outcomes among all assessed immune cell populations [24]. A 2026 study by Luo et al. shows that infiltration of SPP1^+^ neutrophils predicts early recurrence and adverse outcomes in hepatocellular carcinoma patients [25]. Thus, both high levels of circulating neutrophils and TANs are linked to poor prognosis in cancer patients, suggesting a pro‐tumor role of neutrophils in human cancer [12].

The earliest evidence for the pro‐tumor role of neutrophils is presented in a rodent study published by Welch et al. in 1989, which shows that neutrophils from tumor‐bearing rats enhanced lung metastases when co‐injected intravenously with cancer cells [26]. In the recent decade, preclinical in vivo animal studies and clinical data‐driven studies have revealed a variety of mechanisms by which neutrophils promote tumor progression, undermine anti‐tumor immunity, and contribute to therapeutic resistance. A dominant tumor‐promoting mechanism involves neutrophil extracellular traps (NETs), which are web‐like structures of chromatin DNA and granule proteins extruded from neutrophils during a unique form of cell death called NETosis [3]. Park et al. reported that cancer cells actively induce NET formation that supports metastasis [27]. NET‐associated proteases such as neutrophil elastase (NE) and matrix metalloproteinases (MMPs) degrade extracellular matrix barriers and awaken dormant tumor cells, thereby accelerating metastatic outgrowth [28]. Interestingly, chemotherapy can induce NETs, which then awaken dormant tumor cells [29], and surgery can also induce NETs to promote metastasis through reprogramming lipid metabolism of cancer cells [30]. NETs can promote cancer cell migration and invasion via engaging CCDC25 on cancer cells [31, 32] and via EGFR/ERK signaling [33]. Tumor‐derived cytokines such as Chi3l1 and CXCR1/2 agonists stimulate NET formation to foster T cell exclusion [34] and impaired cytotoxicity of T cells and natural killer (NK) cells [35], whereas degradation of NETs restores CD8^+^ T cell activity and improves immune checkpoint blockade efficacy [36].

In addition to NET‐mediated effects, neutrophils directly suppress anti‐tumor immunity and confer resistance to T cell‐based immunotherapy. Neutrophils can inhibit the function of T cells through expression of programmed death‐ligand 1 (PD‐L1) [37] and production of reactive oxygen species (ROS) [38], nitric oxide [39], arginase‐1 [40], and prostaglandin E_2_ (PGE_2_) [41]. Distinct neutrophil subsets, including the urokinase‐type plasminogen activator receptor (PLAUR)^+^ neutrophils [42] and Ly6G^high^ populations [43], promote CD8^+^ T cell dysfunction or death and drive anti‐PD‐1 resistance. Tumor‐derived arachidonic acid [44] and oncogenic factors such as AKR1B10 [45] reprogram neutrophils through metabolic and epigenetic mechanisms, including histone lactylation [45], to enhance immune evasion. In parallel, histone lactylation in tumor cells increases their CXCL1 expression, which amplifies neutrophil recruitment to the tumor and immune escape [46]. Hypoxia‐driven EGR1 signaling remodels neutrophils toward immunosuppressive phenotypes that impair anti‐tumor T cell responses [47]. In the liver metastatic niche, expansion of neutrophils directly inhibits the expansion and cytotoxic function of chimeric antigen receptor (CAR)‐T cells [48].

Tumor‐ and host‐derived local and systemic cues actively reprogram neutrophils into specialized pro‐tumor states. Local signals influence how neutrophils behave upon entering tissues, while systemic signals affect neutrophil differentiation in the bone marrow, influencing their phenotypes and functions as they circulate and enter different tissues. Signals within the tumor tend to dominate over systemic effects, as multiple recent studies have shown. According to a 2024 study by Ng et al., all neutrophils that enter the tumor tissue, regardless of their initial maturation state, will undergo deterministic reprogramming and converge into a shared terminal phenotype in a glycolytic and hypoxic niche in the tumor core [49]. This convergent phenotype is marked by pro‐angiogenic features and favors tumor growth [49]. Another study discovered that neutrophils in this shared tumor state produce CCL3 to promote tumor growth [50]. In breast cancer, tumor‐activated macrophages recruit neutrophils to form physical contact with tumor cells, which increases tumor cell proliferation and invasion and promotes angiogenesis [51]. Likewise, THY1^+^ cancer stem cells internalize neutrophil‐derived mitochondria to adopt a pseudohypoxic state, thereby driving metastasis [52]. Host and environmental factors further shape these tumor‐promoting programs: microbiome dysbiosis drives neutrophil recruitment and mesothelial transition to facilitate peritoneal metastasis [53], and intratumoral bacteria fosters an immunosuppressive tumor microenvironment marked by neutrophil accumulation in head and neck cancer [54].

Systemic effects of tumors on neutrophil functions are not to be overlooked, as these have a particular influence on metastasis. In both mice and humans, neutrophils under the influence of cancer growth increase NET production, promoting the establishment of organ‐specific metastatic niches, including in the brain [55] and liver [56]. Additionally, chronic stress can increase systemic NET formation across tissues, thereby promoting metastatic dissemination [57]. The exact signals from tumors vary by tumor type, but often include G‐CSF [58], CXCL1 [59], IL‐6 [58], IL‐1β [60], and other chemokines. These chemokines usually originate from cancer cells or surrounding stroma, but can also come from immune cells, such as innate lymphoid cells, which produce TNF‐like ligand 1A to induce expansion of tumor‐promoting neutrophil populations [61].

### 2.2 Anti‐Tumor Functions of Neutrophils

Despite a plethora of evidence supporting the pro‐tumor nature of neutrophils, these immune cells have also been reported to exhibit intrinsic anti‐tumor activity without therapeutic intervention. A 1975 study by Clark et al. represents the earliest evidence for the anti‐tumor role of neutrophils, showing that human neutrophils are cytotoxic against cancer cells in vitro [62]. A central anti‐tumor mechanism of neutrophils is direct tumor cell killing, frequently mediated by reactive oxygen species (ROS) and myeloperoxidase (MPO) [62, 63, 64]. Neutrophils also directly trigger apoptosis of bone metastatic prostate cancer cells via STAT5 suppression [65] and mediate antibody‐dependent cellular cytotoxicity (ADCC) via trogoptosis in the presence of tumor‐binding antibodies [66]. Beyond direct killing, neutrophils can attenuate tumor progression by limiting tumor angiogenesis and growth in an endogenous interferon‐β (IFN‐β)‐dependent manner [67] and debriding hypoxic tumor cells in early‐stage PTEN‐deficient uterine cancer [68]. Importantly, neutrophils can also enhance anti‐tumor adaptive immunity by promoting type 1 polarization of unconventional T cells [69], stimulating T cell proliferation and IFN‐γ production [70], and differentiating into an antigen‐presenting state in early‐stage human lung cancer [71]. Similarly, Ly6E^high^ neutrophils induced by tumor‐intrinsic STING (stimulator of interferon genes) activity augment cytotoxic T cell responses and sensitize tumors to anti‐PD‐1 therapy [72]. Studies also show that neutrophils are required for successful T cell‐based immunotherapy [73, 74], which indirectly manifests the ability of neutrophils to help fight cancer.

Another group of studies showcase the potential of neutrophils to perform anti‐tumor functions after intervention or reprogramming. Transforming growth factor‐β (TGF‐β) blockade [75] or IFN‐β [76, 77] polarizes neutrophils from a pro‐tumor phenotype into an anti‐tumor phenotype. Similarly, super‐low dose endotoxin trains neutrophils to adopt a potent immune‐enhancing phenotype and reduce tumor burden [78], and maintained neutrophilia induced by granulocyte colony‐stimulating factor (G‐CSF) increases tumor necrosis and suppresses pancreatic tumor growth [79]. Inhibition of intrinsic negative regulators in neutrophils such as Tollip [80], STAT3 [81], and CXCR4 [82] enhances neutrophil costimulatory and antigen‐presenting functions, promotes CD8^+^ T cell or NK cell functions, and reduces tumor growth and metastasis. Notably, a combination of tumor necrosis factor (TNF), CD40 agonist, and tumor‐binding antibody activates neutrophils to infiltrate and eradicate tumors and prevent metastasis [83, 84]. Similarly, CD40 agonist in combination with anti‐PD‐L1 restores the anti‐tumor activity in neutrophils by inducing their responses to IFNs [85]. Moreover, leucine activates the antigen‐presenting program in neutrophils that further elicits T cell responses and boosts anti‐PD‐1 immunotherapy [86], while IL‐36γ‐armored CAR‐T cells reprogram neutrophils into tumoricidal, antigen‐presenting states that amplify endogenous T cell responses [87].

### 2.3 Dual Functions of Neutrophils in Cancer

Intriguingly, neutrophils are shown to exhibit both anti‐tumor and pro‐tumor activity in the same study, with their net impact shaped by interactions with other immune cells and the tumor microenvironment. Neutrophils inhibit the lung metastasis of breast cancer in NK cell‐deficient mice but promote it in NK cell‐competent mice in the same tumor models [88]. Ex vivo co‐culture experiments in the same study show that neutrophils alone kill tumor cells, but they suppress the tumor cytotoxicity of NK cells, as the ROS produced by neutrophils is toxic to both NK and cancer cells [88]. Similarly, NK cells restrain a tumor‐promoting neutrophil phenotype through IFN‐γ; in their absence, neutrophils upregulate VEGF‐A, enhance angiogenesis, and drive tumor progression [89]. In glioblastoma, neutrophils induce MPO‐dependent ferroptosis and tumor necrosis, yet this cytotoxicity against tumor cells paradoxically fuels tumor aggressiveness and correlates with poor prognosis [64]. Together, these studies underscore the context‐dependent dual functions of neutrophils in cancer.

### 2.4 Beyond the Binary: Multiple States of Neutrophils in Cancer

Recent single‐cell RNA‐sequencing studies have revealed an unprecedented level of heterogeneity within the neutrophil population that transcends the traditional pro‐/anti‐tumor binary view of neutrophils [49]. A global neutrophil atlas in mice NeuMap revealed that neutrophils occupy defined functional hubs and diverge into interferon‐responsive, angiogenic, immunosuppressive, or quiescent states along distinct trajectories that are reshaped in cancer [90]. Across 17 cancer types, single‐cell transcriptome analyses of human neutrophils identified at least ten transcriptionally distinct states, including an antigen‐presenting program associated with favorable prognosis and improved immunotherapy response [86]. In non‐small cell lung cancer, high‐resolution atlases uncovered diverse tissue‐resident human neutrophil subpopulations with context‐dependent plasticity linked to PD‐L1 therapy resistance [91]. Multimodal single‐cell profiling of human colorectal cancer revealed spatial organization and functional niches of neutrophils and discovered a neutrophil subset with antigen‐presenting properties [92]. In human liver cancer, single‐cell analyses stratified the tumor immune microenvironment into five subtypes and identified CCL4^+^ and PD‐L1^+^ TANs that correlate with poor prognosis [93]. Similarly, cervical cancer progression is marked by stage‐specific neutrophil states, culminating in SPP1^+^/GBP1^+^/ELOVL5^+^ tumor‐promoting phenotypes [94]. These studies establish TANs as a highly diverse compartment composed of multiple transcriptional and functional states.

## 3 Neutrophils as a Therapeutic Target for Cancer

Despite being the most abundant immune cell population in circulation and one of the most abundant in various tumor tissues, neutrophils have gained limited attention as potential therapeutic targets for cancer compared to other immune cells such as T cells [1, 83]. This can be partially attributed to the notion that neutrophils are evolutionarily programmed to fight infectious pathogens rather than to fight or promote cancer [95]. The short lifespan of neutrophils, their heterogeneity, and the limited understanding of their role in cancer also present challenges to targeting these immune cells for cancer treatment [1, 5, 96]. Nonetheless, the past decade has witnessed a surge of new research that further elucidates the tumor‐modulating mechanisms of neutrophils and explores innovative strategies to harness neutrophils against cancer, suggesting a turning point for the era of neutrophil‐based cancer immunotherapy. In this section, we review major neutrophil‐targeting therapeutic strategies that have recently been developed and evaluated in the preclinical stage, while highlighting representative clinical trials. Therapeutic strategies targeting NETs in cancer have been comprehensively reviewed elsewhere [97] and therefore will not be discussed in detail here.

### 3.1 Blockade of Neutrophil Recruitment to Tumor

**FIGURE 1:**
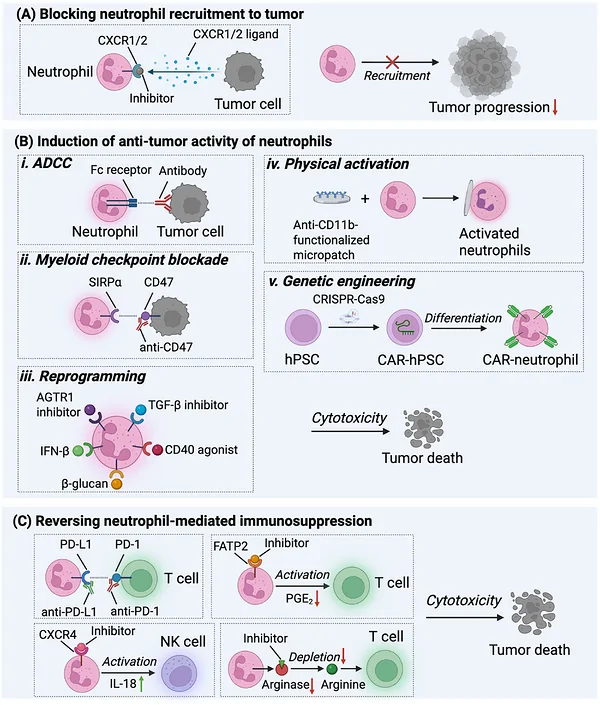
Therapeutic strategies that target neutrophils for cancer treatment. (A) Blockade of neutrophil recruitment to tumor. Inhibition of neutrophil chemokine receptor CXCR1/2 can block neutrophil recruitment and attenuate tumor progression [99, 104]. (B) Induction of anti‐tumor activity of neutrophils. (i) Tumor‐targeting antibodies can opsonize cancer cells and induce Antibody‐dependent cellular cytotoxicity (ADCC) by neutrophils [66]. (ii) Blockade of myeloid checkpoints, such as the signal‐regulatory protein alpha (SIRPα)/CD47 “don't eat me” signaling pathway, enables neutrophils to directly kill cancer cells [119]. (iii) Reprogramming neutrophils with TGF‐β blockade [75], IFN‐β [76, 77], angiotensin II type 1 receptor (AGTR1) antagonism [120], CD40 agonism [83], or β‐glucan [121] can boost the tumor‐killing ability of neutrophils. (iv) Physical activation by attaching backpack‐like polymer micropatches to the surface of neutrophils can transform neutrophils into an anti‐tumor state [127]. (v) Genetic engineering of human pluripotent stem cells (hPSCs) can generate chimeric antigen receptor (CAR)‐neutrophils with potent, antigen‐specific cytotoxicity against solid tumors [129]. (C) Reversing neutrophil‐mediated immunosuppression on T cells and NK cells. Blockade of the PD‐L1/PD‐1 signaling axis [137] and blockade of the fatty acid transport protein 2 (FATP2) [41] facilitate activation and tumor cytotoxicity of T cells. Similarly, Blockade of CXCR4 facilitates activation and tumor cytotoxicity of NK cells [82]. Inhibition of neutrophil‐secreted arginase restores extracellular arginine availability, thereby enhancing anti‐tumor responses of T cells and NK cells [138]. Created with BioRender.com.

Since neutrophils are widely reported to engage in pro‐tumor activity after recruitment to the tumor site, blockade of such recruitment presents a straightforward therapeutic strategy that can prevent neutrophils from becoming TANs and facilitating tumor progression (Figure 1A). Neutrophils express the chemokine receptors CXCR1 and CXCR2, which can sense tumor‐secreted chemokines such as interleukin‐8 (IL‐8) and mediate neutrophil chemotaxis and thus recruitment toward the tumor [98]. Many murine studies have shown that inhibition of the CXCR2 signaling axis attenuates neutrophil recruitment to tumors, reduces tumor burden, and improves responses to chemotherapy or T cell‐based immunotherapy [99, 100, 101, 102, 103, 104, 105, 106, 107, 108, 109, 110, 111, 112, 113, 114]. Two CXCR2 inhibitors (MK‐7123 and AZD‐5069) and three CXCR1/2 inhibitors (Reparixin, Ladarixin, and SX‐682) have entered clinical trials for cancer treatment; several have demonstrated good safety and tolerability, although clear efficacy has yet to be established [1, 115]. CXCR1 and CXCR2 are also expressed by monocytes, NK cells, mast cells, and epithelial cells [116, 117], so additional confounding effects from blocking both pro‐tumor cells and anti‐tumor cells may be present.

### 3.2 Induction of Anti‐Tumor Activity of Neutrophils

While inhibiting the recruitment of neutrophils to tumors can mitigate their pro‐tumor effect, alternative strategies seek to unleash or enhance their intrinsic cytotoxicity to maximize their anti‐tumor potential. The first approach is to administer tumor‐targeting antibodies to induce ADCC where neutrophils express Fc receptors that can recognize and kill antibody‐opsonized cancer cells [66, 118] (Figure 1Bi). Another approach is blockade of myeloid checkpoints expressed on neutrophils, such as the signal‐regulatory protein alpha (SIRPα)/CD47 “don't eat me” signaling pathway, which enables neutrophils to directly kill cancer cells [66, 119] (Figure 1Bii). Moreover, neutrophil reprogramming with TGF‐β blockade [75], IFN‐β [76, 77], angiotensin II type 1 receptor (AGTR1) antagonism [120], or β‐glucan [121] can increase the tumor‐killing ability of neutrophils (Figure 1Biii). A study by Linde et al. developed a neutrophil‐activating cocktail consisting of TNF, a CD40 agonist, and a tumor‐binding antibody to induce potent cytotoxic neutrophils for treatment in several cancer models [83].

Recently, several innovative engineering approaches have sought to mobilize or reprogram neutrophils for cancer treatment. One major approach leverages neutrophils as delivery vehicles for nanomedicine due to their natural tropism toward tumors, a topic comprehensively reviewed elsewhere [122, 123]. More recent studies have further demonstrated the versatility of this strategy. For example, neutrophil membrane‐coated nanoparticles improved chemotherapy efficacy on liver metastasis of colorectal cancer [124]; neutrophils were employed as “Trojan horses” to transport nanoparticles across the blood‐brain barrier for glioblastoma treatment [125]; and neutrophil “hitchhiking” was identified as a key mechanism driving sustained nanoparticle redistribution to tumors and spleen [126]. A second strategy physically activates neutrophils using drug‐free surface engineering: attachment of backpack‐like polymer micropatches to the surface of neutrophils polarized them toward an anti‐tumor phenotype and synergized with immune checkpoint blockade in mouse models of melanoma and glioblastoma [127, 128] (Figure 1Biv). Moreover, adoptive transfer of genetically engineered chimeric antigen receptor (CAR)‐neutrophils derived from human pluripotent stem cells has demonstrated potent, antigen‐specific cytotoxicity against solid tumors including glioblastoma [129], prostate cancer [130], and melanoma [131], and has been further integrated with nanodrug delivery as a combination therapy for glioblastoma [132] (Figure 1Bv). Chang et al. produced CAR‐neutrophils in vivo by direct genetic programming of primary neutrophils using engineered extracellular vesicle‐ or lipid nanoparticle‐based RNA delivery platforms for glioma treatment. This approach demonstrated anti‐tumor efficacy in humanized mouse models and favorable safety in canine studies [133]. Recently, the UK‐based biotechnology company LIfT BioSciences developed Immunomodulatory Alpha Neutrophils (IMANs), the world's first off‐the‐shelf, allogeneic, and long‐lived neutrophil‐based cell therapy generated from induced pluripotent stem cells or CD34^+^ hematopoietic stem cells [134]. This pioneering therapy has shown potential tumor‐killing activity in preclinical in vivo and ex vivo models [135] and is expected to enter a Phase 1 clinical trial for cervical cancer and head and neck cancer in early 2027 [136]. Together, these engineering approaches position neutrophils as versatile platforms for targeted drug delivery, material‐based activation, and adoptive cell therapy in cancer.

### 3.3 Reversing Neutrophil‐Mediated Immunosuppression

As mentioned previously, neutrophils can inhibit the anti‐tumor functions of T cells and NK cells. Hence, another therapeutic strategy is to reverse neutrophil‐mediated immunosuppression on these cytotoxic immune cells (Figure 1C). Blockade of neutrophil interaction with immune checkpoints expressed on T cells, such as the PD‐L1/PD‐1 signaling pathway, helps remove the inhibitory effect on anti‐tumor T cell responses [137]. Similarly, blockade of the fatty acid transport protein 2 (FATP2) on neutrophils can reduce PGE_2_ production and thus facilitate T cell activation [41]. Moreover, blockade of CXCR4 on neutrophils activates NK cells by upregulating secretion of interleukin‐18 (IL‐18) [82]. Arginase, another key immunosuppressive molecule secreted by neutrophils, depletes available extracellular arginine that T cells and NK cells rely on for effector functions [40], and thus inhibition of arginase can improve T cell and NK cell responses. A phase I clinical trial of an arginase inhibitor is underway for treatment of solid tumors (NCT02903914) [138]. Thus, beyond direct tumor cell killing, neutrophils can, following therapeutic intervention, instruct other immune cells such as T cells and NK cells to execute tumor‐killing effector functions.

## 4 Microphysiological Systems (MPS): Complex In Vitro Models

**FIGURE 2:**
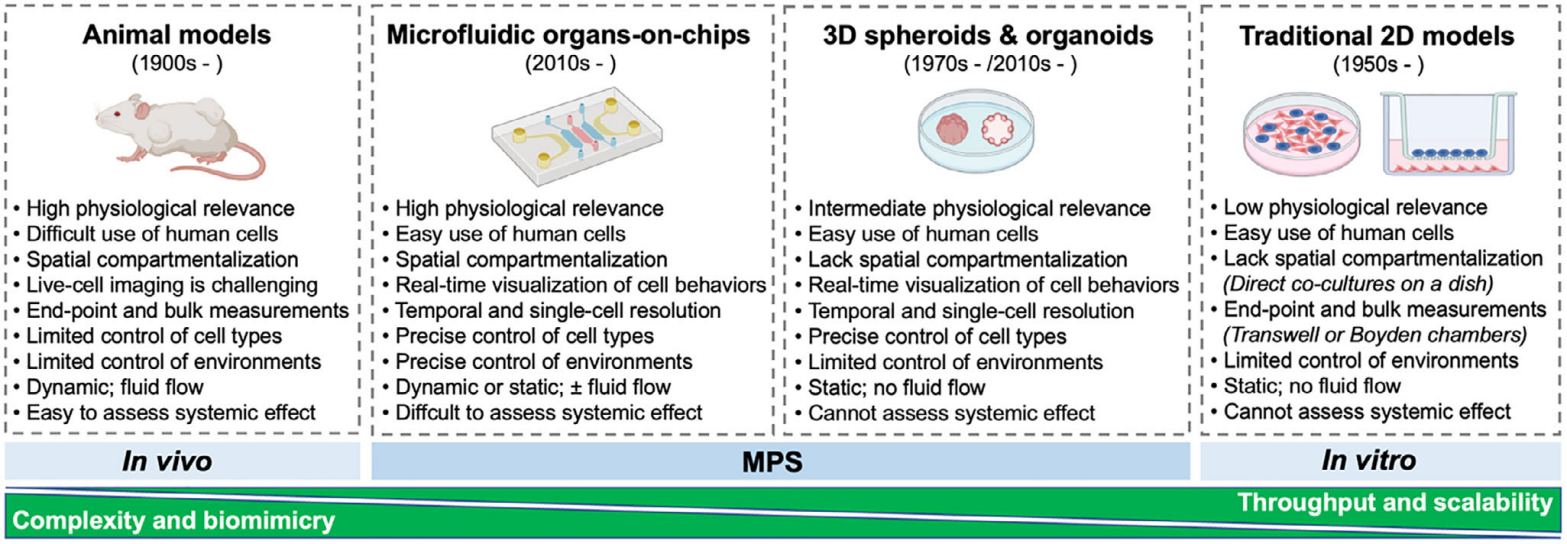
Comparisons between different preclinical models for cancer immunology research. MPS are more physiologically relevant than traditional in vitro models and easily enable real‐time visualization and monitoring of human cell behaviors and interactions with single‐cell resolution, which can be challenging to attain in in vivo animal models. MPS help bridge the gap between in vitro and in vivo models by balancing physiological complexity and biomimicry with experimental throughput and scalability. The historical period when the model system was first used or started to gain popularity is indicated below each category. Created with BioRender.com.

Animal models have the longest history among all preclinical model systems and have been used for cancer immunology research since the 1900s [139, 140]. Consistent with this long‐standing reliance on animals, research on neutrophils in cancer to date has predominantly used in vivo models, supplemented in some cases by in silico analyses of clinical transcriptomic datasets of human cancers. In contrast, experimental wet‐lab studies directly using human neutrophils remain limited, in part due to their inherent fragility and short lifespan ex vivo [141]. Researchers have used traditional in vitro models, such as two‐dimensional (2D) monolayer (co‐)cultures [53, 62, 81, 142, 143, 144, 145, 146, 147] and transwell chambers [145, 148, 149, 150, 151], to investigate human neutrophil‐cancer interactions. Some studies examined human neutrophil chemotaxis toward tumor‐derived signals [145, 149]. Others reported that human neutrophils promoted cancer cell migration, invasion, and proliferation [142, 144, 146, 148, 149, 150]. In contrast, several studies demonstrated the anti‐tumor effects of human neutrophils, including direct cytotoxicity and suppression of tumor cell proliferation [62, 81, 145, 147]. However, these in vitro models are often too simplistic to reflect the conditions of the in vivo tumor microenvironment, and findings obtained from such systems may have limited physiological relevance. The recent emergence of microphysiological systems (MPS) has made it possible to study interactions between human neutrophils and cancer in physiologically relevant in vitro settings. According to a 2023 workshop report by leaders in the field of MPS, the definition of MPS encompasses all complex in vitro models that are not traditional 2D cell cultures, including well plate‐based three‐dimensional (3D) culture models represented by spheroids and organoids and microfluidics‐based organ‐on‐a‐chip models [15]. In this section, we introduce two major types of MPS, namely 3D spheroid and organoid models and microfluidic organ‐on‐a‐chip models, and discuss their respective benefits as useful tools for investigating the role of human neutrophils in cancer (Figure 2).

### 4.1 3D Spheroid and Organoid Models

First introduced in 1971 by Sutherland et al. [152], spheroids refer to multicellular, dense, and usually homogeneous 3D constructs [16]. In the context of cancer, tumor spheroids are 3D aggregates of cancer cells that recapitulate important features of solid tumors, such as cell‐cell and cell‐extracellular matrix (ECM) interactions, multilayered structure (an outer proliferating layer, a middle quiescent layer, and an inner necrotic core), gradients of metabolic, nutrient, and oxygen diffusions, clinically relevant resistance to chemotherapy, and in vivo‐like gene expression patterns [16, 153, 154]. These features make spheroids more physiologically relevant than 2D cultures for modeling cancer biology (Figure 2). With immortalized cancer cell lines as the most common cell source, tumor spheroids are mainly produced through the following two techniques: the hanging drop method and ultra‐low attachment plates, where the absence of contact with adherent surfaces forces cancer cells to adhere to each other and form aggregates spontaneously [16, 155]. The size of spheroids can be controlled by adjusting the initial cell seeding density, and heterotypic spheroids can be created by co‐seeding cancer cells with stromal cells such as fibroblasts [16, 154]. Owing to these desirable features, as well as their ease of fabrication, spheroids have become one of the most widely used 3D models for studying tumor biology and for high‐throughput screening of anticancer drugs [16, 154].

Organoids are self‐organized multicellular 3D constructs derived from adult, embryonic, or induced pluripotent stem cells that resemble key structural and functional features of an organ or tissue of interest [156]. Modern organoid technology emerged through two seminal advances. In 2008, Yoshiki Sasai's group demonstrated the self‐organization of polarized cortical tissues from embryonic stem cells, providing one of the earliest examples of pluripotent stem cell‐derived organoids [157]. In 2009, Hans Clevers's group established long‐term cultures of adult intestinal stem cells that self‐organized into crypt‐villus structures, laying the foundation for the widespread adoption of organoid technology [158]. Since these pioneering studies, organoid models have rapidly expanded to encompass numerous human organs, including the brain, lung, kidney, and liver [156]. Organoid cultures are established in extracellular matrix (ECM)‐like hydrogels such as Matrigel and collagen and are guided by defined combinations of signaling molecules and growth factors at different stages to promote differentiation into specific cell types of interest [16, 154]. Tumor organoids are usually generated from primary tumor cells in resected tissues or biopsies directly from cancer patients, and thus they preserve the cellular heterogeneity and genetic programming of the native tumor and mimic the natural development of a tumor more faithfully than spheroids [16, 159]. In summary, the major distinction between spheroids and organoids is that tumor spheroids are grown from immortalized cancer cell lines, are easier to generate, but less complex in structure, while tumor organoids are grown from primary tumor samples and require more maintenance, but more closely resemble the original tumor.

### 4.2 Microfluidic Organ‐on‐a‐Chip Models

Microfluidic organ‐on‐a‐chip models represent a class of bioengineered, sophisticated in vitro platforms that use human cells to mimic the structures and functions of tissues and organs in microfluidic devices [15, 160]. These microfluidic devices contain small chambers or channels of defined geometry which fluids such as cell suspensions and culture medium can flow through [19, 160]. Organ‐on‐a‐chip systems can be either static (no flow) or dynamic (with flow). These systems can incorporate 3D spheroids, organoids, or other tissue engineering structures to recapitulate key aspects of the tumor microenvironment, usually involving the use of hydrogel to mimic the extracellular matrix [161, 162, 163, 164, 165]. The microfluidic devices are typically fabricated using standard microfabrication techniques: photolithography is first performed to create a master mold by adding pre‐designed geometric patterns to a silicon wafer, followed by soft lithography, in which a liquid polymeric material is cast onto the mold and cured to produce a solid, rubber‐like replica [19, 164]. The most commonly used material is polydimethylsiloxane (PDMS) due to its flexibility, low cost, gas permeability, biocompatibility, and optical transparency, which makes imaging easy [19, 164].

Microfluidic organ‐on‐a‐chip models hold several advantages over 3D in vitro models and in vivo animal models when it comes to cancer immunology research (Figure 2). These systems allow the spatial confinement and compartmentalization of distinct cell types such as immune cells and cancer cells using specialized structures such as porous membranes [166], microposts (micropillars) [167], phaseguides [168], and microgrooves [169], and these features are not available in direct co‐cultures in 2D or 3D formats on well plates [170]. Unlike most in vivo animal models, organ‐on‐a‐chip models accommodate live imaging of human cells easily and enable direct visualization and quantification of cellular behaviors and interactions with single‐cell resolution in real time [17]. Dynamic fluid flow can be applied and controlled in microfluidic devices to create shear stress and mimic blood circulation and interstitial flow, which is superior to static conditions in 2D and 3D cultures on well plates [19, 160]. By generating stable chemical concentration gradients [22] and applying dynamic mechanical forces [166], microfluidic devices also enable more precise control over the biochemical and mechanical environments that cells are exposed to than other in vitro and in vivo models [17, 160]. Overall, MPS, including spheroids, organoids, and microfluidic organ‐on‐a‐chip models, help bridge the gap between traditional in vitro and in vivo models by balancing physiological complexity and biomimicry with experimental throughput and scalability.

### 4.3 Benefits of MPS for Studying the Role of Neutrophils in Cancer

MPS offer several important advantages for studying neutrophil biology in cancer. One major advantage is that these systems can be constructed using human cells, thereby overcoming fundamental species‐specific differences between human and murine neutrophils that limit the translational relevance of animal models [14]. For example, the relative abundance of lymphocytes and neutrophils differs substantially between humans and mice: human blood is neutrophil‐rich (50%–70% neutrophils), while laboratory mouse blood is lymphocyte‐rich (10%–25% neutrophils) [171]. The typical neutrophil marker Ly‐6G is only expressed in mice but not in humans, and some chemokines (such as IL‐8) and their receptors (such as CXCR1) that affect neutrophil recruitment to tumors are present in humans but lack direct orthologs in mice [14]. Unlike human neutrophils, mouse neutrophils lack the expression of FcαRI (CD89), a key Fc receptor that induces neutrophil effector functions including ROS production, NET formation, and ADCC [14]. A study by Jones et al. shows that human neutrophils respond to chemoattractants with substantially higher levels of activation and migration than their rodent counterparts [172]. Likewise, Cui et al. show that human neutrophils are more capable of lysing cancer cells at baseline than murine neutrophils [173]. Thus, MPS‐based research on human neutrophils in cancer can help overcome limitations of previously mentioned murine studies and reduce the translational gap between animal models and human diseases.

Beyond enabling the use of human cells, MPS provide several unique experimental capabilities for investigating neutrophil biology in cancer. The three‐dimensional architecture of spheroids, organoids, and microfluidic organ‐on‐a‐chip models more faithfully recapitulates the tumor microenvironment than conventional 2D cultures, allowing neutrophils to migrate through extracellular matrix barriers and infiltrate solid tumor tissues in a manner that more closely resembles the in vivo setting. Microfluidic platforms enable independent control of parameters including spatial organization of distinct cell types, biochemical gradients, mechanical cues, and fluid flow, thereby allowing systematic dissection of how individual factors in the tumor microenvironment regulate neutrophil function. Importantly, the optical accessibility of MPS supports high‐resolution live‐cell imaging, making it possible to quantitatively analyze diverse neutrophil behaviors—including migration, direct neutrophil‐cancer cell interactions, tumor infiltration, and tumor cytotoxicity—with single‐cell resolution in real time (see Section 8.2.1 for details). Collectively, these features of MPS can provide mechanistic insights into neutrophil behavior in cancer that are difficult to obtain using conventional in vitro or animal models.

### 4.4 Toward Regulatory Approval of MPS

The past five years have witnessed multiple milestones pushing forward the regulatory acceptance of MPS as alternative tools to animals for preclinical drug testing [174, 175, 176, 177, 178, 179] (Table 1). The US Food and Drug Administration (FDA) approved the first Investigational New Drug (IND) application filed in 2020 using MPS‐generated efficacy data for two rare neuromuscular diseases, leading to the start of a clinical trial (NCT04658472) in April 2021 [180]. In December 2022, former US President Biden signed into law the FDA Modernization Act 2.0, adding “organ chips and MPS” to the list of nonclinical tests [181, 182]. In September 2024, the human Liver‐Chip developed by leading organ‐on‐a‐chip company Emulate was accepted into the FDA's Innovative Science and Technology Approaches for New Drugs (ISTAND) pilot program for predicting drug‐induced liver injury (DILI) in humans [175]. In April 2025, the FDA announced the plan to phase out the requirement of animal testing for monoclonal antibodies and other drugs [183], while the National Institutes of Health (NIH) announced in July 2025 that it will no longer award funding to new grant proposals solely relying on animal models, encouraging alternative methodologies such as MPS and in silico models [184, 185]. In September 2025, the NIH announced the establishment of the Standardized Organoid Modeling Center, the first dedicated national research center for organoids in the US, aimed at reducing reliance on animal models [186, 187]. In October 2025, the FDA approved the world's first IND application for a cancer immunotherapy treatment based solely on organ‐on‐a‐chip data without animal testing [188, 189]. In December 2025, the US Senate passed the FDA Modernization Act 3.0, which strengthens the adoption of MPS as an alternative to animal models for drug testing [190]. On March 18, 2026, the FDA issued a draft guidance supporting the validation of new approach methodologies (NAMs), including MPS and in silico models, as alternatives to animal testing to accelerate drug development using human‐centric data [191]. On April 1, 2026, organ‐on‐a‐chip devices from Emulate were sent into space aboard NASA's Artemis II mission to study how human bone marrow tissues respond to radiation and microgravity, providing insights into astronaut immune function [192]. On June 15, the NIH announced the launch of the Office of Research Innovation, Validation, and Application (ORIVA) to speed the development and use of human‐based research technologies and NAMs that can better reflect human biology than animals, including organoids, organs‐on‐chips, and computational models [193].

**TABLE 1:** Major milestones leading to regulatory acceptance of MPS in the US.

Time	Milestone
Apr 2021	Efficacy data from MPS supported the authorization of a clinical trial.
Dec 2022	FDA Modernization Act 2.0 signed into law, adding “organ‐chips and MPS” to the list of nonclinical tests.
Sept 2024	Human Liver‐Chip accepted in FDA's ISTAND for prediction of DILI.
Apr 2025	FDA plans to phase out animal testing requirement for monoclonal antibodies.
July 2025	NIH announced that new funding opportunities would no longer be issued exclusively for animal models and would also support human‐focused approaches and NAMs.
Sept 2025	NIH announced establishment of Standardized Organoid Modeling Center.
Oct 2025	FDA cleared an IND amendment supported by human vascularized organoid/MPS efficacy data without traditional animal efficacy/POC studies for the combination therapy.
Dec 2025	US Senate passed FDA Modernization Act 3.0.
Mar 2026	FDA released draft guidance on alternatives to animal testing in drug development.
Apr 2026	Organ‐chips flown on NASA's Artemis II to study space biology.
Jun 2026	NIH launched new office to advance human‐based research and reduce animal use.

## 5 Application of 3D Spheroid and Organoid Models to Studying Human Neutrophil‐Cancer Interactions

Spheroids and organoids are the most popular 3D models for investigating tumor biology in vitro. In this section, we summarize the in vitro studies of human neutrophil‐tumor interactions using 3D spheroid and organoid models based on well plates (Table 2).

**TABLE 2:** Summary of 3D models to study neutrophil‐cancer interactions.

Type of 3D model	Neutrophil source	Cancer type (cell line)	Key finding/significance	Reference
Tumor spheroid	Healthy donors’ blood	Lung cancer (A549)	Neutrophil infiltration increases tumor growth rate; pro‐tumor effect of neutrophils. **CXCR2 inhibition as a therapeutic strategy**.	[194]
	Healthy donors’ blood	Breast cancer (MDA‐MB‐231; BT549; BT474; M4)	The more aggressive TNBC induces greater neutrophil recruitment than the less aggressive ER+ variant; pro‐tumor effect of neutrophils.	[195]
	Healthy donors’ blood	Colorectal cancer (LS174T; HT29)	NETs physically protect tumor cells from T cell and NK cell cytotoxicity; pro‐tumor effect of neutrophils. **NET clearance or inhibition as a therapeutic strategy**.	[35]
	Healthy donors’ blood	HNSCC (patient‐derived)	NETs inhibit the tumor cytotoxicity and tumor infiltration of NK cells; pro‐tumor effect of neutrophils. **NET clearance or inhibition as a therapeutic strategy**.	[196]
	Human pluripotent stem cells	Glioblastoma (U87MG)	CAR‐neutrophils show potent tumor infiltration and tumor cytotoxicity. **CAR‐neutrophils as a cell therapy**.	[129]
	Human pluripotent stem cells	Glioblastoma (U87MG)	R‐SiO_2_‐TPZ NP‐loaded CAR‐neutrophils show better tumor cytotoxicity than unloaded CAR‐neutrophils. **Drug‐loaded NPs + CAR‐neutrophils as a combination therapy**.	[132]
Tumor organoid (patient‐derived)	Healthy donors’ blood	Colorectal cancer	Polarization of neutrophils toward a pro‐tumor state by colorectal tumor is KRAS‐dependent; pro‐tumor effect of neutrophils.	[92]
	Patient blood	Colorectal cancer	AKR1B10 in cancer cells reprograms neutrophils into an immunosuppressive state; pro‐tumor effect of neutrophils. **AKR1B10 inhibition as a therapeutic strategy**.	[45]
	Induced pluripotent stem cells or hematopoietic stem cells	Pancreatic cancer and other solid tumors	Immunomodulatory alpha neutrophils (IMANs) show potent, antigen‐independent killing across multiple solid tumor types. **IMANs as a cell therapy**.	[135]
	Patient blood	NSCLC	KRT10 binds NETs to promote spread of NSCLC tumor cells in brain organoids. Metastasis‐promoting effect of NETs.	[55]

Abbreviations: HNSCC, head and neck squamous cell carcinoma; NP, nanoparticle; NSCLC, non‐small cell lung cancer; TPZ, tirapazamine.

*Note*: Neutrophil‐targeting therapeutic strategies or anti‐tumor effects of neutrophils reported in these studies are bolded.

### 5.1 3D Spheroid Models

**FIGURE 3:**
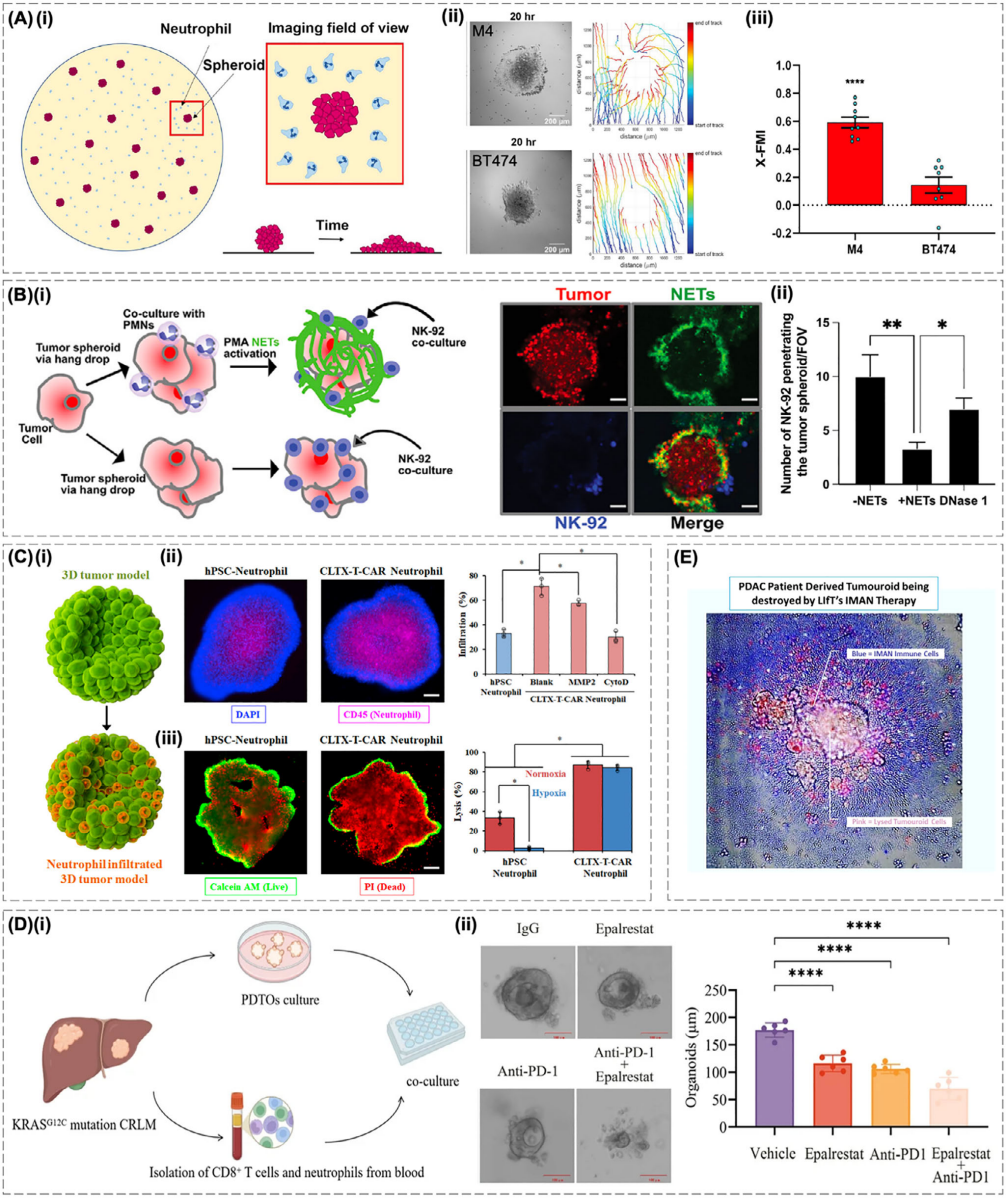
3D spheroid and organoid models to investigate human neutrophil‐tumor interactions. (A) (i) A co‐culture system to measure neutrophil migration toward different types of breast tumor spheroids on a glass‐bottom well plate. (ii) Representative images of M4 (more aggressive) and BT474 (less aggressive) tumor spheroids and tracks of neutrophil migration over 20 h via time‐lapse imaging. (iii) Directionality of neutrophil migration toward spheroids was quantified as forward migration index in the x‐direction (x‐FMI). Adapted with permission from ref [195], Copyright 2021 Frontiers. (B) (i) Neutrophils were co‐cultured with head and neck tumor spheroids (red), induced with PMA to release NETs (green), and then co‐cultured with NK‐92 cells (blue). (ii) NETs inhibit NK cell penetration of tumor spheroids, which is reversed by treatment with DNase I to digest NETs. Adapted with permission from ref [196], Copyright 2024 Elsevier. (C) (i) A co‐culture model of glioblastoma tumor spheroids with chimeric antigen receptor (CAR)‐neutrophils generated from human pluripotent stem cells (hPSCs). (ii) CLTX‐T‐CAR neutrophils show higher infiltration into tumor spheroids than hPSC neutrophils, the wild‐type control. (iii) CLTX‐T‐CAR‐neutrophils induce greater lysis of tumor spheroids than hPSC neutrophils. Adapted with permission from ref [129], Copyright 2022 Elsevier. (D) (i) Tumor organoids grown from liver metastases of colorectal cancer patients with KRAS^G12C^ mutation were co‐cultured with autologous CD8^+^ T cells and neutrophils isolated from peripheral blood of these patients. (ii) A combination of AKR1B10 inhibitor epalrestat and anti‐PD‐1 attenuates tumor organoid growth more than either treatment alone. Adapted with permission from ref [45], Copyright 2026 Springer Nature. (E) Immunomodulatory Alpha Neutrophils (IMANs), a neutrophil‐based cell therapy developed by LIfT BioSciences, show potent killing of tumoroids derived from pancreatic ductal adenocarcinoma (PDAC) patients. Adapted with permission from LIfT BioSciences news release, May fourth 2023, ref. [197].

The following studies used 3D tumor spheroids to investigate the role of human neutrophils in cancer in a more biomimetic environment than traditional 2D cultures. Tazzyman et al. co‐cultured primary human neutrophils with tumor spheroids from the A549 human lung cancer cell line and found that neutrophil infiltration into tumor spheroids increased their growth rate, showing a pro‐tumor effect of neutrophils. The researchers also found that CXCR2 antagonist AZ10397767 reduced neutrophil infiltration into A549 spheroids, suggesting CXCR2 inhibition as a potential therapeutic strategy [194]. SenGupta et al. co‐cultured primary human neutrophils with tumor spheroids from human triple negative breast cancer (TNBC) or estrogen receptor‐positive (ER+) cell lines on a 24‐well plate (Figure 3A). Using time‐lapse imaging of the co‐culture system, the authors found that the more aggressive TNBC induced greater neutrophil recruitment than the less aggressive ER+ variant [195]. Teijeira et al. co‐cultured LS174T or HT29 colorectal tumor spheroids with first human neutrophils and then CD8^+^ T cells or NK cells. The authors found that NETs that formed around tumor cells can block their physical contact with T cells and NK cells, thereby inhibiting the cytotoxicity of these immune cells against tumor cells. Treatment with DNase I, which digests the extracellular DNA in NETs restored T cell and NK cell cytotoxicity, suggesting NET clearance or inhibition as a therapeutic strategy [35]. Likewise, Babatunde et al. performed a tri‐culture of neutrophils, head and neck tumor spheroids, and NK cells to study the effect of neutrophils on the anti‐tumor activity of NK cells (Figure 3B). The researchers showed that tumor spheroids recruited both neutrophils and NK cells, and that NETs inhibited NK cell infiltration into and cytotoxicity against tumor spheroids. This inhibitory effect was reversed by treatment with DNase I, showing the promise of anti‐NETs therapy [196]. Chang et al. developed CAR‐neutrophils from human pluripotent stem cells and showed their potent infiltration into and cytotoxicity against glioblastoma tumor spheroids (Figure 3C). The researchers also found that compared to control neutrophils, CAR‐neutrophils maintained high expression levels of anti‐tumor markers even under hypoxia, which mimics the in vivo tumor microenvironment [129]. In a later study, the same research group loaded their CAR‐neutrophils with chemotherapy‐containing SiO2 nanoparticles and showed superior cytotoxicity of these upgraded neutrophils against glioblastoma tumor spheroids compared to original CAR‐neutrophils or nanoparticles alone [132]. Together, these neutrophil‐tumor spheroid co‐culture systems enabled the evaluation of various neutrophil‐based therapeutic strategies for cancer, including CXCR2 inhibition, NET clearance, and stem cell engineering.

### 5.2 3D Organoid Models

The following studies used patient‐derived tumor organoids to examine the role of human neutrophils in cancer as a more clinically relevant model than cell line‐derived tumor spheroids. Marteau et al. co‐cultured primary human neutrophils with conditioned media from colorectal cancer patient‐derived tumor organoids of different mutational backgrounds and found that KRAS‐mutant tumors prolonged neutrophil survival and polarized neutrophils into a tumor‐promoting, T cell‐suppressing phenotype [92]. To confirm the role of AKR1B10 in reprogramming neutrophils toward an immunosuppressive phenotype in the liver metastasis of colorectal cancer with KRAS^G12C^ mutation, Li et al. grew tumor organoids from liver metastases of colorectal cancer patients and co‐cultured them with autologous neutrophils and CD8^+^ T cells from peripheral blood of the same patients (Figure 3D). The study showed a synergy between AKR1B10 inhibition and anti‐PD‐L1 therapy in suppressing tumor organoid growth, indicating AKR1B10 as a potential therapeutic target [45]. Moreover, IMANs, the neutrophil‐based cell therapy developed by LIfT BioSciences, demonstrated potent antigen‐independent killing of patient‐derived tumor organoids of various cancer types, including pancreatic cancer (Figure 3E). These findings highlight the potential of IMANs to target antigen escape variants that drive metastasis, which accounts for the majority of cancer‐related deaths [135]. Lastly, a study by Chen et al. used brain organoids instead of tumor organoids to study the role of KRT10 in the brain metastasis of non‐small cell lung cancer (NSCLC). KRT10 is a NET‐binding protein expressed by NSCLC tumor cells. The authors co‐cultured NSCLC patient‐derived tumor cells with neutrophils in cerebral organoids and found that KRT10 knockdown reduced the spread of NSCLC tumor cells within the organoids in the presence of NETs, suggesting a metastasis‐promoting effect of NETs [55]. Collectively, these studies highlight the power of the organoid technology to reveal novel tumor‐promoting molecular mechanisms of neutrophils and test neutrophil‐based therapies in a clinically relevant manner.

## 6 Application of Microfluidic Organ‐on‐a‐Chip Models to Studying Human Neutrophil‐Cancer Interactions

The earliest microfluidics‐based study of human immune cell‐cancer interactions was published by Huang et al. in 2009, where the researchers developed a microfluidic device to monitor the behaviors of metastatic breast cancer cells and macrophages embedded in 3D hydrogels in adjacent chambers [198]. Ever since then, microfluidic organ‐on‐a‐chip models of various shapes and sizes have been developed to investigate the interactions of cancer cells with stromal cells, including endothelial cells and fibroblasts, and immune cells, including T cells, NK cells, macrophages, dendritic cells, and neutrophils, and to evaluate cancer immunotherapy [162, 163, 164, 165, 199, 200, 201]. Since 2018 [202], microfluidic systems have been utilized to model the involvement of human neutrophils in multiple stages of tumor progression, including neutrophil recruitment to the primary tumor site, neutrophil‐cancer interactions within the tumor microenvironment, and intravascular neutrophil‐cancer interactions that facilitate metastatic dissemination. In this section, we review these organ‐on‐a‐chip studies based on the stage of tumor progression being modeled (Table 3). We also mention the application of these models to the evaluation of neutrophil‐based cancer immunotherapies when applicable.

**TABLE 3:** Summary of microfluidic organ‐on‐a‐chip models to study neutrophil‐cancer interactions.

Stage of tumor progression	Neutrophil source	Cancer type (cell line)	Key finding/significance	Reference
Neutrophil recruitment to primary tumors	Patient blood	Cervical cancer (SiHa)	Recreation of patient‐specific neutrophil recruitment to the TME incorporating donor‐matched cervical fibroblasts and NET release. Patient‐on‐a‐chip.	[203]
	Healthy donors’ blood	Prostate cancer (C4‐2)	Regulatory role of cancer‐associated fibroblasts in neutrophil recruitment to tumor. Potential to predict patient‐specific cancer‐immune response.	[204]
	Differentiation of HL‐60 human cell line	Pancreatic cancer (PANC‐1)	CXCR2 inhibition reduces neutrophil migration to tumor spheroids in velocity, directionality, and displacement. **CXCR2 inhibition as a therapeutic strategy**.	[205]
	Differentiation of HL‐60 human cell line	Pancreatic cancer (PANC‐1)	Pro‐tumor neutrophils show greater migration to cancer cells and higher motility after migration than anti‐tumor neutrophils.	[206]
Neutrophil‐cancer interactions in tumors	Healthy donors’ blood	Breast cancer (MDA‐MB‐231); HNSCC (patient‐derived)	Cancer cells trigger migration, clustering, and altered metabolic activity of neutrophils. Neutrophils restrict cancer cell migration and invasion and induce their apoptosis. **Anti‐tumor effect of neutrophils**.	[196]
	Healthy donors’ blood	Ovarian cancer (OVCAR‐3)	NET release by recruited neutrophils promoted tumor invasion in ECM; pro‐tumor effect of neutrophils. **NET inhibition as a therapeutic strategy**.	[207]
	Healthy donors’ blood	Brain‐metastatic breast cancer (MDA‐MB‐231)	Brain‐metastatic breast cancer upregulates CXCR2 ligands to increase neutrophil recruitment and NET release; pro‐tumor effect of neutrophils. **CXCR2 inhibition as a therapeutic strategy**.	[208]
	Differentiation of HL‐60 human cell line	Pancreatic cancer (PANC‐1)	CXCR2 inhibition attenuates tumor invasion and proliferation via blocking direct contact of tumor with neutrophils; pro‐tumor effect of neutrophils. **New anti‐tumor mechanism of CXCR2 inhibition as a therapeutic strategy**.	[205]
	Differentiation of HL‐60 human cell line	Pancreatic cancer (PANC‐1)	“Behaviorome” profiling of anti‐tumor and pro‐tumor neutrophil subtypes. **Anti‐tumor polarization as a therapeutic strategy**.	[206]
	Differentiation of HL‐60 human cell line	Pancreatic cancer (PANC‐1)	Anti‐tumor neutrophils enhance migration and tumor cytotoxicity of NK cells. **Reversing neutrophil‐mediated immunosuppression as a therapeutic strategy**.	[209]
Neutrophil‐metastatic cancer cell interactions in blood vessels	Differentiation of HL‐60 human cell line	Breast cancer (MCF‐7; MDA‐MB‐231)	Development of a tool for generating CTC‐neutrophil clusters, which can be used for metastasis studies.	[210]
	Mouse neutrophil‐conditioned medium	Breast cancer (MDA‐MB‐231)	Neutrophil‐secreted factors activate endothelial cells to promote cancer cell extravasation. Metastasis‐promoting effect of neutrophils.	[211]
	Healthy donors’ blood	Melanoma (A375; A375‐MA2); Breast cancer (MDA‐MB‐231)	Role of systemic inflammation in metastasis. Inflamed neutrophils promote cancer cell extravasation via chemotactic confinement. Metastasis‐promoting effect of neutrophils.	[202]
	Human buffy coat	Breast cancer (MDA‐MB‐231)	Platelets collude with neutrophils to promote cancer cell extravasation. Metastasis‐promoting effect of neutrophils. Inhibition of platelet integrin β3 as a therapeutic strategy.	[212]
	Human buffy coat	Breast cancer (MDA‐MB‐231)	Extravasated neutrophils induced death of metastatic breast cancer cells in the bone. **Anti‐tumor effect of neutrophils**.	[147]

Abbreviations: CTC, circulating tumor cells; ECM, extracellular matrix; HNSCC, head and neck squamous cell carcinoma; NET, neutrophil extracellular traps; TME, tumor microenvironment.

*Note*: Neutrophil‐targeting therapeutic strategies or anti‐tumor effects of neutrophils reported in these studies are bolded.

### 6.1 Modeling Neutrophil Recruitment to the Tumor Tissue

**FIGURE 4:**
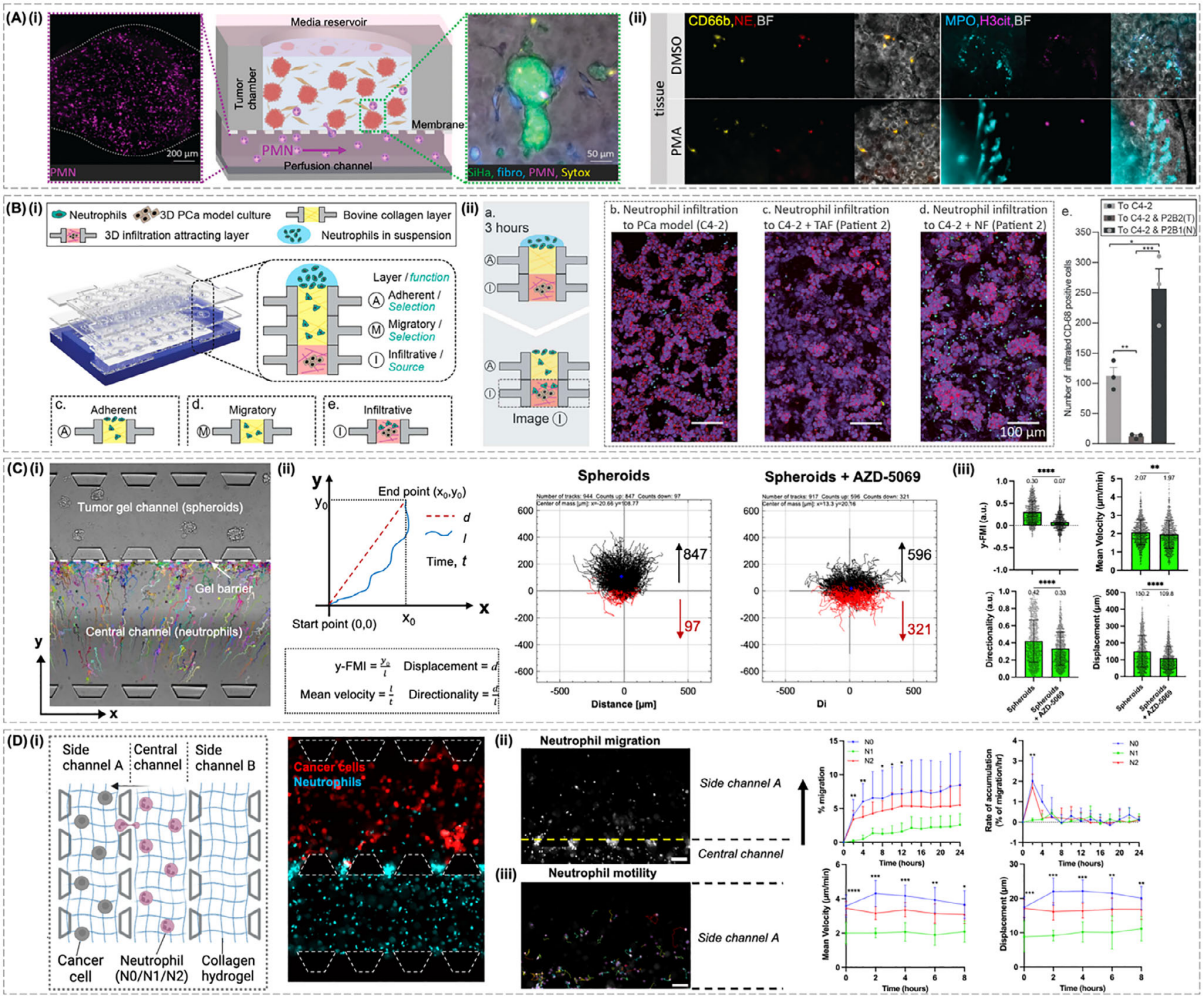
Microfluidic organ‐on‐a‐chip systems to model neutrophil recruitment to tumors. (A) A cervical cancer‐on‐a‐chip model recreates patient‐derived neutrophil recruitment to tumor tissue. (i) The device contains a tumor tissue chamber with hydrogel‐embedded SiHa tumor spheroids and fibroblasts and a perfusion channel for introducing neutrophils (PMN). (ii) Immunofluorescence images showing the presence of neutrophils (CD66b and NE) and formation of NETs (MPO and H3cit) in the tumor tissue chamber. Adapted with permission from ref. [203], Copyright 2023 Wiley Online Library. (B) A reconfigurable microscale device examines the effect of cancer‐associated fibroblasts on neutrophil infiltration toward prostate tumor tissue with spatiotemporal manipulations. (i) The device consists of three vertical layers for separating neutrophils based on their infiltrative ability with the tumor tissue as the source of attraction. (ii) Patient‐specific fibroblasts from benign and cancerous regions of the prostate affect neutrophil infiltration to tumor differently. Green = neutrophils, red = EpCAM (cancer cells), blue = nuclei. Adapted with permission from ref. [204]., copyright 2021 Oxford University Press. (C) A microfluidic device is used to study the effect of CXCR2 inhibitor AZD‐5069 on neutrophil migration to pancreatic cancer. (i) Time‐lapse imaging captures neutrophils (color‐coded) in the central channel migrating toward collagen‐embedded pancreatic tumor spheroids in the tumor gel channel. (ii) Trajectory plots showing single‐cell trajectories of neutrophil migration, from which detailed parameters of motility can be quantified. (iii) AZD‐5069 reduces y‐forward migration index (y‐FMI), mean velocity, directionality, and displacement of neutrophil migration. Adapted with permission from ref. [205], Copyright 2024 Springer Nature. (D) (i) A microfluidic model is used to compare the migration of different neutrophil subtypes (naïve, anti‐tumor, and pro‐tumor) to pancreatic cancer cells in a 3D collagen matrix and the motility post‐migration. (ii) Pro‐tumor neutrophils show greater migration to cancer cells than anti‐tumor neutrophils, quantified as the percentage of migrated neutrophils and the rate of accumulation over time. (iii) Pro‐tumor neutrophils show higher motility after migration than anti‐tumor neutrophils in terms of mean velocity and displacement. Adapted with permission from ref. [206], Copyright 2025 Royal Society of Chemistry.

A few microfluidics‐based studies seek to examine the process of neutrophil recruitment into the tumor tissue as an early stage of tumor progression. A cervical cancer‐on‐chip model used 3D tumor spheroids from the SiHa human cell line and patient‐derived fibroblasts embedded in extracellular matrix‐mimicking dextran hydrogel to build the tumor tissue chamber. The model recapitulated the recruitment of patient‐derived primary human neutrophils from the perfusion channel across the porous membrane into the tumor chamber and their functional ability to generate NETs [203] (Figure 4A). Yu et al. developed a reconfigurable microscale platform that vertically stacks three separable collagen hydrogel‐filled layers to stratify neutrophils based on their ability to migrate and infiltrate toward the prostate tumor tissue at the bottom layer (Figure 4B). Using primary human neutrophils from healthy donors and fibroblasts from prostate cancer patients, this platform revealed that cancer‐associated fibroblasts (CAFs) limit neutrophil infiltration compared to fibroblasts from benign prostate tissues in a patient‐specific manner [204]. Shao et al. built a microfluidic device that encapsulated pancreatic tumor spheroids from the PANC‐1 human cell line in a collagen hydrogel to mimic the pancreatic tumor tissue. The researchers seeded neutrophils into the adjacent chamber in culture medium and performed time‐lapse live imaging to capture and quantify the temporal dynamics of neutrophil migration toward tumor spheroids with single‐cell resolution (Figure 4C). They found that small‐molecule CXCR2 antagonist AZD‐5069 reduced the forward migration index, velocity, directionality, and displacement of neutrophil migration [205], which agrees with the finding of previous in vivo studies showing the ability of CXCR2 inhibition to reduce neutrophil recruitment to pancreatic tumor tissue in mice [99, 100, 101]. A similar microfluidic device was later utilized to quantitatively compare the migration of different neutrophil subtypes to pancreatic cancer cells in 3D collagen matrix and their motility post‐migration (Figure 4D). The authors differentiated the HL‐60 human leukemia cell line into a neutrophil‐like state, which is the most commonly used model for primary human neutrophils, and polarized the cells into either an anti‐tumor or pro‐tumor subtype. Using time‐lapse imaging and single‐cell tracking, the authors found that pro‐tumor neutrophils showed greater migration to cancer cells and higher post‐migration motility than anti‐tumor neutrophils [206].

### 6.2 Modeling Neutrophil‐Cancer Interactions Within the Tumor Microenvironment

**FIGURE 5:**
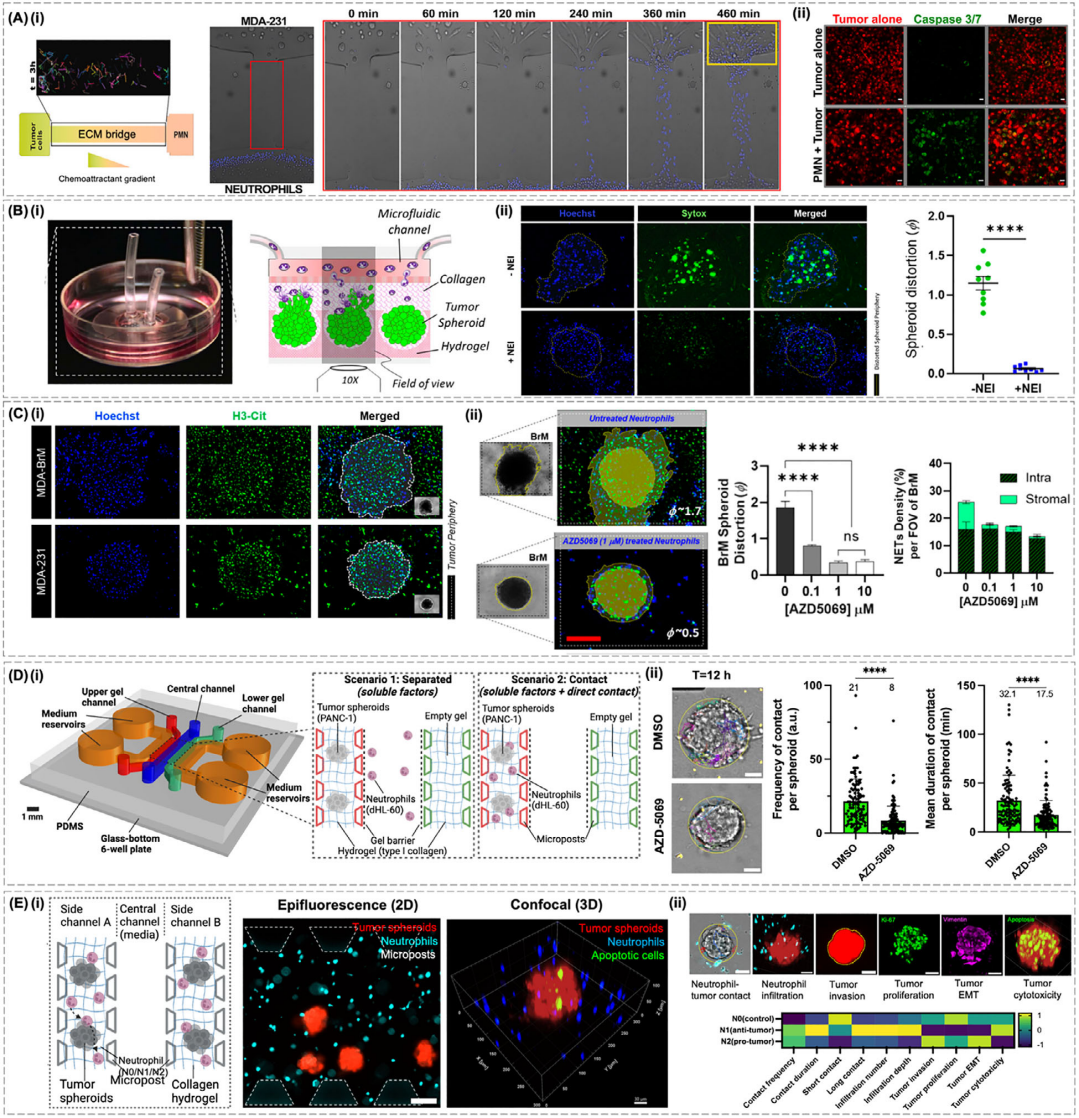
Microfluidic organ‐on‐a‐chip systems to model neutrophil‐cancer interactions within the tumor microenvironment. (A) (i) An under‐oil microfluidic platform captures neutrophil (PMN) migration across an extracellular matrix bridge toward tumor cells and the ensuing interactions with them. (ii) Primary human neutrophils triggered the caspase 3/7 apoptotic pathway in tumor cells. Adapted with permission from ref. [196], Copyright 2024 Elsevier. (B) (i) An ovarian cancer‐on‐a‐chip system captures neutrophil migration toward tumor spheroids, NET formation, and tumor spheroid invasion in a collagen matrix. (ii) Inhibition of NETs reduce neutrophil‐induced distortion and invasion of tumor spheroids, suggesting NETs as a therapeutic target. Blue = neutrophil nuclei, green = NETs. The dashed yellow line circles the tumor spheroid boundary. Adapted with permission from ref. [207], Copyright 2021 IOP Publishing Ltd. (C) (i) The same device in (B) was repurposed to study neutrophil interactions with brain‐metastatic breast tumor spheroids. Blue = neutrophil nuclei, green = NETs. (ii) CXCR2 inhibition via small‐molecule antagonist AZD‐5069 reduces NET formation and tumor spheroid invasion. Adapted with permission from ref. [208], Copyright 2022 MDPI. (D) (i) A microfluidic device enables the creation of both “separated” and “contact” scenarios to tease out the important role of direct contact in the tumor‐promoting mechanisms of neutrophils and the tumor‐suppressive mechanisms of CXCR2 inhibition. (ii) CXCR2 inhibitor AZD‐5069 reduces both the frequency and duration of direct contact between neutrophils and tumor spheroids, measured using time‐lapse images. Adapted with permission from ref. [205], Copyright 2024 Nature Portfolio. (E) (i) A microfluidic device models the interactions between different neutrophil subtypes (naïve, anti‐tumor, and pro‐tumor) and pancreatic tumor spheroids in 3D collagen matrix. (ii) Neutrophil subtypes exhibit distinct behaviors including contact with tumor, infiltration into tumor, tumor cytotoxicity, and influences on tumor progression in terms of invasion, proliferation, and epithelial‐mesenchymal transition (EMT), quantified and summarized in a heatmap. Adapted with permission from ref. [206], Copyright 2025 Royal Society of Chemistry.

The following studies not only recapitulated the recruitment of neutrophils to the tumor tissue but also examined their subsequent interactions with cancer cells after recruitment, providing insight into both neutrophil and tumor behaviors. Babatunde et al. built an under‐oil microfluidic platform where healthy donor‐derived primary human neutrophils from one chamber were allowed to migrate through an extracellular matrix bridge toward cancer cells in another chamber and then directly interact with them (Figure 5A). The researchers observed that cancer cells triggered migration, swarming‐like cluster formation, ROS production, and NET release of neutrophils, while optical metabolic imaging revealed elevated metabolic activity in neutrophils in direct contact with cancer cells compared to non‐contact neutrophils. They also found that neutrophils restricted the migration and invasion of breast cancer cells and induced their apoptosis, indicating an anti‐tumor, cytotoxic function of neutrophils [196]. An ovarian cancer‐on‐a‐chip model demonstrated that primary human neutrophils migrated into the tumor chamber containing tumor spheroids embedded in a collagen gel matrix and generated NETs that stimulated collective tumor cell invasion into the surrounding matrix, showing a pro‐tumor effect of neutrophils (Figure 5B). Pharmacological inhibition of NET formation attenuated tumor invasion, suggesting NETs as a potential therapeutic target [207]. The same microfluidic device was used by Safarulla et al. to investigate the role of neutrophil chemokine receptor CXCR2 in brain metastasis of breast cancer (Figure 5C). The researchers found that pharmacological inhibition of CXCR2 reduced neutrophil recruitment toward brain‐metastatic breast tumor spheroids and subsequent NET formation and suppressed tumor invasion, suggesting CXCR2 blockade as a potential therapeutic strategy [208]. Another study revealed the important role of direct contact in the tumor‐promoting mechanisms of neutrophils and the tumor‐suppressive mechanisms of CXCR2 inhibition by using a microfluidic device to spatially control the contact between neutrophils and tumor spheroids and measure the invasion and proliferation of tumor spheroids (Figure 5D). The researchers also quantified the temporal dynamics of neutrophil‐tumor spheroid contact using time‐lapse images and found that CXCR2 antagonist AZD‐5069 reduced both the frequency and duration of such contact [205]. Shao et al. established a microfluidic co‐culture model to profile the interactions between different neutrophil subtypes (naïve, anti‐tumor, and pro‐tumor) and pancreatic tumor spheroids in the same 3D collagen matrix (Figure 5E).

The authors found that anti‐tumor neutrophils engaged in longer contact with tumor spheroids and infiltrated greater into tumor spheroids than pro‐tumor neutrophils. Anti‐tumor neutrophils also attenuated the invasion, proliferation, and epithelial‐mesenchymal transition (EMT) of tumor spheroids compared to naive and pro‐tumor neutrophils, although tumor cytotoxicity was limited in all three subtypes [206]. In a later study, NK cells were incorporated into the same microfluidic device to investigate the dual effect of neutrophils on NK cell behavior. The researchers found that NK cells showed preferential migration toward anti‐tumor neutrophils over pro‐tumor neutrophils, and that anti‐tumor neutrophils restored NK cell cytotoxicity against tumor spheroids while pro‐tumor neutrophils suppressed it [209]. These two studies suggest polarization or reprogramming of neutrophils as a potential immunotherapy strategy for cancer, either through direct anti‐tumor functions or by boosting anti‐tumor functions of NK cells in the tumor microenvironment.

### 6.3 Modeling Neutrophil‐Cancer Interactions in Blood Vessels

**FIGURE 6:**
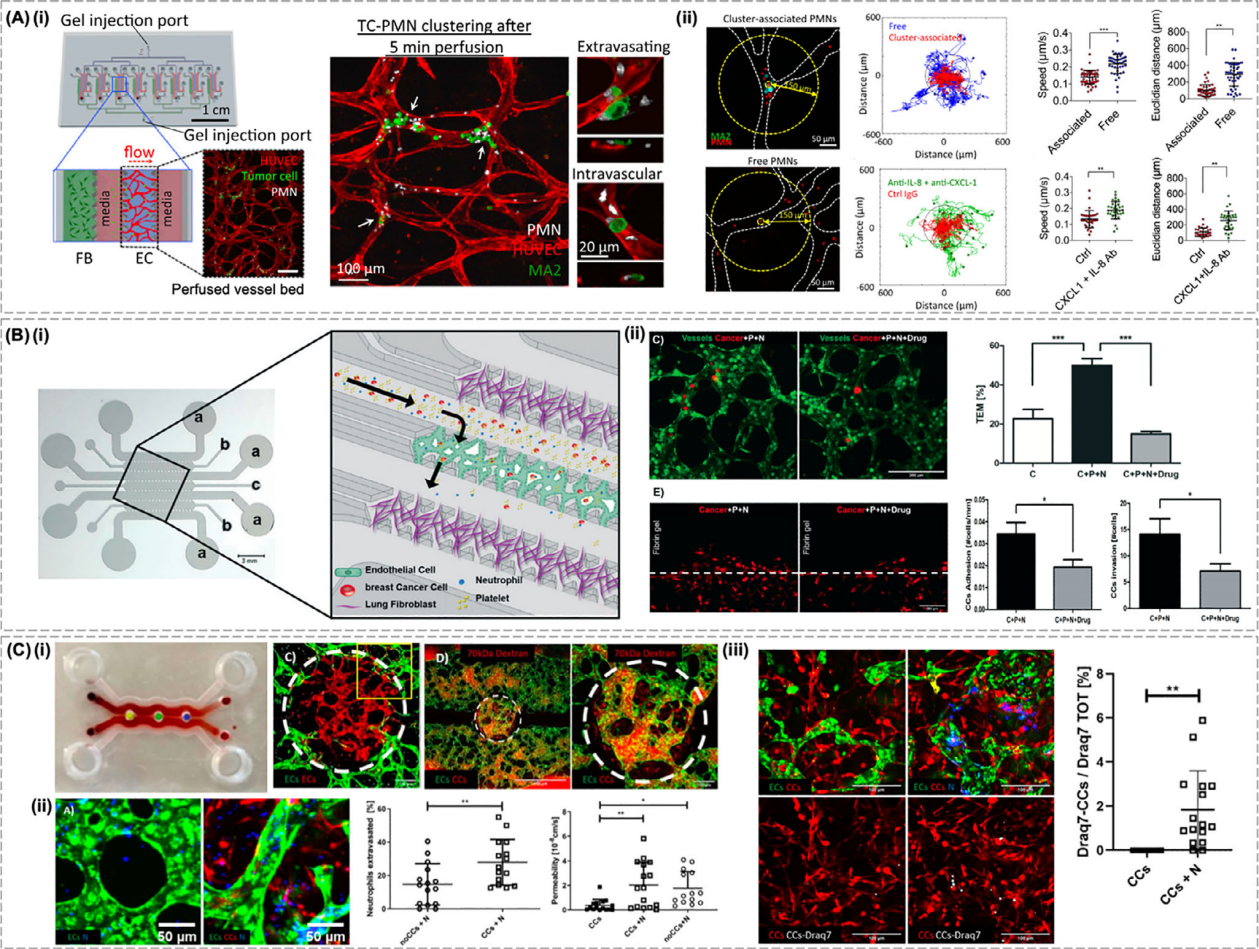
Microfluidic organ‐on‐a‐chip systems to model neutrophil‐cancer cell interactions in blood vessels. (A) (i) A human microvasculature on‐a‐chip system was built using human umbilical vein endothelial cells (HUVECs) and fibroblasts (FB) embedded in fibrin gels under fluid flow. Neutrophils (PMN) and MA2 tumor cells were perfused together into the vessel beds to capture their intravascular interactions. (ii) Time‐lapse confocal imaging enables tracking of neutrophil migration with single‐cell resolution and quantification of neutrophil speed and Euclidean distance under different conditions. Adapted with permission from ref. [202], Copyright 2018 National Academy of Sciences. (B) (i) An “early metastatic niche”‐on‐a‐chip model sought to decipher the crosstalk among cancer cells, neutrophils, platelets, and endothelial cells during metastatic dissemination through blood vessels. (ii) Platelets (P) and neutrophils (N) promoted cancer cell extravasation. An anti‐platelet drug (integrin β3 inhibitor) reduced cancer cell adhesion, trans‐endothelial migration (TEM), and invasion, showing an anti‐metastatic effect. Adapted with permission from ref. [212], Copyright 2021 Royal Society of Chemistry. (C) (i) A microfluidic device mimics the travel of circulating neutrophils through blood vessels into the bone colonized by metastatic breast cancer cells. (ii) The presence of cancer cells in the bone microenvironment increased neutrophil extravasation, which led to a higher permeability of the endothelial cell barrier. (iii) Neutrophils introduced into the vascular network induced the death of cancer cells in the bone microenvironment as indicated by the Draq7 dead cell stain. ECs = endothelial cells, CCs = cancer cells. N = neutrophils. Adapted with permission from ref. [147], Copyright 2022 Elsevier.

Microfluidic organ‐on‐a‐chip systems have also been developed to model interactions between neutrophils and circulating tumor cells (CTCs) in blood vessels, which happens after cancer cells leave the primary tumor site and before they extravasate to colonize the secondary site to complete metastasis. A proof‐of‐concept study by Park et al. presents a tool for the investigation of CTC‐neutrophil clusters as potent mediators of metastasis. The researchers fabricated a droplet microfluidic chip that deterministically encapsulates tumor cells and neutrophils to generate clusters with well‐defined cellular ratios and molecular signatures [210]. One major advantage of microfluidic systems is the ability to induce fluid flow and use human endothelial cells to build vascular structures that mimic blood vessels. Spiegel et al. generated a perfusable, self‐organized microvascular network in a microfluidic device using human umbilical vein endothelial cells (HUVECs) and normal human lung fibroblasts as important supporting cells embedded in a fibrin hydrogel. They treated the microvascular network with mouse neutrophil‐conditioned medium before introducing human breast cancer cells into the lumen of the network. The researchers found that neutrophil‐secreted factors can facilitate protrusion formation and extravasation of intraluminal tumor cells indirectly by priming endothelial cells, highlighting the metastasis‐promoting role of neutrophils [211]. Chen et al. used a similar human microvasculature on‐a‐chip platform and co‐perfused human neutrophils and tumor cells together into the vascular beds to capture their intravascular interactions with high spatiotemporal resolution using time‐lapse confocal microscopy (Figure 6A). They found that inflamed (LPS‐stimulated) neutrophils form aggregates with CTCs and become chemotactically confined by self‐secreted IL‐8 and tumor‐derived CXCL‐1 near these clusters, resulting in localized IL‐8 signaling that disrupts endothelial barrier integrity and enhances tumor cell extravasation. The organ‐on‐a‐chip in this study helped discover a new mechanism by which neutrophil‐mediated systemic inflammation promote metastatic seeding [202]. Crippa et al. developed an “early metastatic niche”‐on‐a‐chip model to better understand the complex crosstalk among cancer cells, leukocytes, platelets, and endothelial cells in the circulation during metastatic dissemination (Figure 6B). The researchers showed that neutrophils and platelets cooperated to enhance extravasation of CTCs and that inhibition of platelet integrin β3 reduced platelet aggregation, restored endothelial junction integrity, and decreased tumor cell extravasation, illustrating the potential of the system for testing anti‐metastatic drugs [212]. In another study, Crippa et al. engineered a breast cancer metastasis to bone (BMtB)‐on‐a‐chip model to investigate the role of circulating neutrophils in the bone colonization of metastatic breast cancer (Figure 6C). Human neutrophils were introduced into a perfusable microvascular network that leads to the bone tissue already colonized by metastatic breast cancer cells. The researchers found that the presence of metastatic tumor seeds in the bone tissue promoted neutrophil extravasation into the bone tissue. Notably, these extravasated neutrophils increased cancer cell death in the bone tissue, highlighting the context‐dependent anti‐tumor functions of neutrophils [147].

## 7 Current Challenges and Future Perspectives

MPS, including spheroids, organoids, and microfluidic organ‐on‐a‐chip models, have shown great promise in modeling human neutrophil‐cancer interactions and evaluating neutrophil‐based cancer immunotherapies. However, this application of MPS remains in its early stages, with significant opportunities for further development. In this section, we first discuss current challenges and limitations associated with using MPS to study human neutrophil functions. We then highlight future applications of MPS for advancing both mechanistic understanding and clinical translation of neutrophil‐based cancer immunotherapies.

### 7.1 Current Challenges

Despite their considerable promise, several challenges currently limit the broader adoption of MPS for studying human neutrophil‐cancer interactions. Primary human neutrophils are highly sensitive and short‐lived cells, and isolation from whole blood may itself induce unintended activation and phenotypic alterations [213]. In addition, donor‐to‐donor variability in primary neutrophils can complicate experimental reproducibility and interpretation. Although patient‐derived tumor organoids can partially recapitulate tumor architecture and heterogeneity, many systems still lack integrated stromal, vascular, and immune components required to fully reproduce the complexity of the tumor microenvironment. Technical limitations associated with microfluidic platforms also remain, including absorption of small hydrophobic drug molecules by PDMS [214] and tradeoffs between physiological complexity and experimental throughput. Furthermore, despite the recent regulatory acceptance of MPS for preclinical drug evaluation (Table 1), widespread implementation of MPS will require increased standardization and validation across different laboratories in the field. Important factors that need to be standardized include cell sources, neutrophil isolation methods, assay timing, culture medium selection, fluid flow conditions, extracellular matrix composition, imaging parameters, quantitative analysis pipelines, and benchmark datasets. Addressing these challenges will be important for improving the robustness, reproducibility, and translational relevance of neutrophil‐focused MPS.

Beyond these technical challenges, several limitations continue to constrain the full potential of MPS for studying neutrophil biology in cancer. Most MPS studies still rely primarily on endpoint measurements rather than real‐time profiling of dynamic neutrophil behaviors. Live‐cell imaging in MPS is rarely integrated with transcriptomic or other multi‐omics analyses to connect neutrophil behaviors with their underlying molecular programs. In addition, relatively few in vitro models incorporate autologous patient‐derived neutrophils and tumor cells, limiting the application of MPS to personalized medicine. Finally, most neutrophil‐focused MPS remain biologically reductionist and contain only a limited number of cell types or a single organ compartment, making it difficult to investigate complex immune crosstalk or systemic responses to therapy. Addressing these limitations will expand the biological capabilities and translational relevance of MPS and enable the future directions discussed in the following section.

### 7.2 Future Perspectives

**FIGURE 7:**
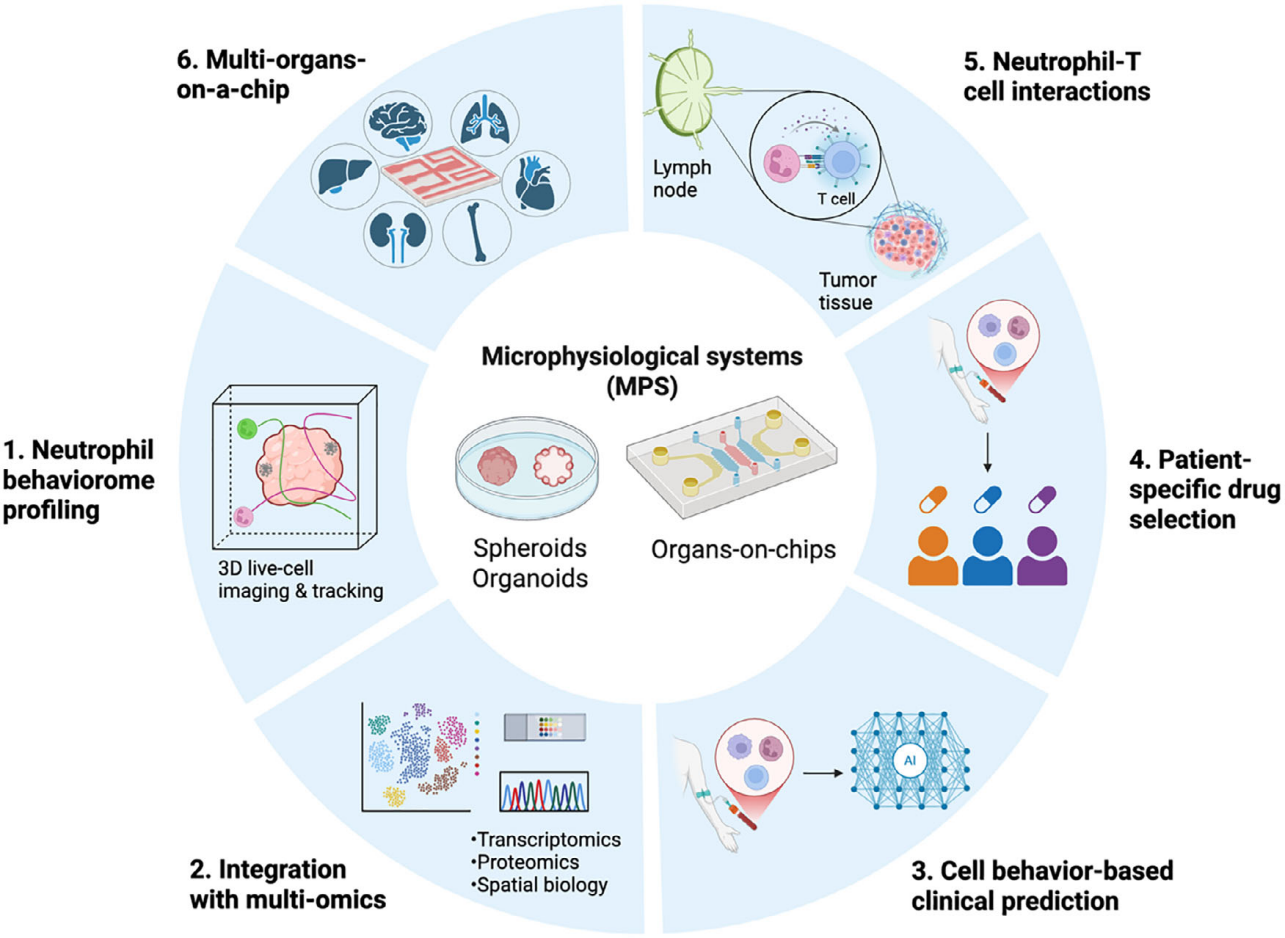
Future applications of MPS to advance neutrophil‐based cancer immunotherapy. (1) MPS‐based profiling of the neutrophil behaviorome in cancer will provide spatiotemporal details of cell‐cell interactions and reveal therapeutic modes of action. (2) Integration of behaviorome profiling with multi‐omics could reveal the molecular drivers behind dynamic neutrophil functions. MPS can push forward personalized medicine by enabling behavior‐based prediction of clinical outcomes (3) and therapy selection (4) using patient‐derived cells. (5) Modeling neutrophil crosstalk with other immune cells such as T cells in tumor‐on‐a‐chip and lymph node‐on‐a‐chip systems will provide a more holistic view of neutrophil biology in cancer. (6) Multi‐organs‐on‐a‐chip systems will enable testing of both the efficacy and systemic toxicity of neutrophil‐based cancer immunotherapy. Created with BioRender.com.

In this subsection, we highlight emerging directions through which MPS can deepen our understanding of neutrophil biology in cancer and facilitate the clinical translation of neutrophil‐based immunotherapies. In particular, MPS‐based profiling of the neutrophil “behaviorome” in cancer can provide spatiotemporal details of cell‐cell interactions and reveal therapeutic modes of action. Integration of behaviorome profiling with multi‐omics approaches could uncover the molecular programs underlying dynamic neutrophil functions. In addition, MPS can expand the scope of personalized medicine by enabling behavior‐based, AI‐guided prediction of clinical outcomes and therapy selection using patient‐derived cells. Finally, modeling neutrophil crosstalk with other immune cells in MPS will provide a more holistic view of the complex roles of neutrophils in cancer, and multi‐organ‐on‐a‐chip systems will enable evaluation of both the efficacy and safety of neutrophil‐based immunotherapy in human‐relevant settings (Figure 7).

### 7.3 MPS‐Based Profiling of the Neutrophil “Behaviorome” in Cancer

Historically, studies using traditional in vitro and in vivo models have been dominated by end‐point measurements of different aspects of neutrophil‐cancer interactions, such as neutrophil migration and infiltration, cancer cell death, and marker expression. In addition to end‐point assays, it is also beneficial to conduct real‐time analysis to capture the temporal dynamics of cellular behaviors. Notably, live‐cell imaging captures the sequence of cellular movements and signaling events over time, providing a wealth of biologically meaningful information that can reveal cellular states, mechanisms of cell‐cell interactions, and modes of action of therapies [18, 215]. The temporal dynamics of neutrophil‐cancer interactions were often missed due to the technical difficulty of performing live‐cell imaging in traditional models. In comparison, one prominent advantage of MPS is that they easily accommodate time‐lapse live‐cell imaging and thus enable real‐time observation of multi‐parametric cellular behaviors with high spatiotemporal resolution [17, 22, 216, 217, 218]. Borrowing from the concept of multi‐omics, here we introduce the term “behaviorome” to denote the complete set of measurable cellular behaviors. Previous studies have used other words and phrases such as high‐level functional outputs, dynamic cellular features, and complex phenotypes to describe similar ideas [18, 219, 220]. Analogous to multi‐omics profiling, we propose that characterization of the neutrophil behaviorome will shed light on neutrophil functional states and tumor‐modulating mechanisms, which can be facilitated by live‐cell imaging in MPS platforms.

**FIGURE 8:**
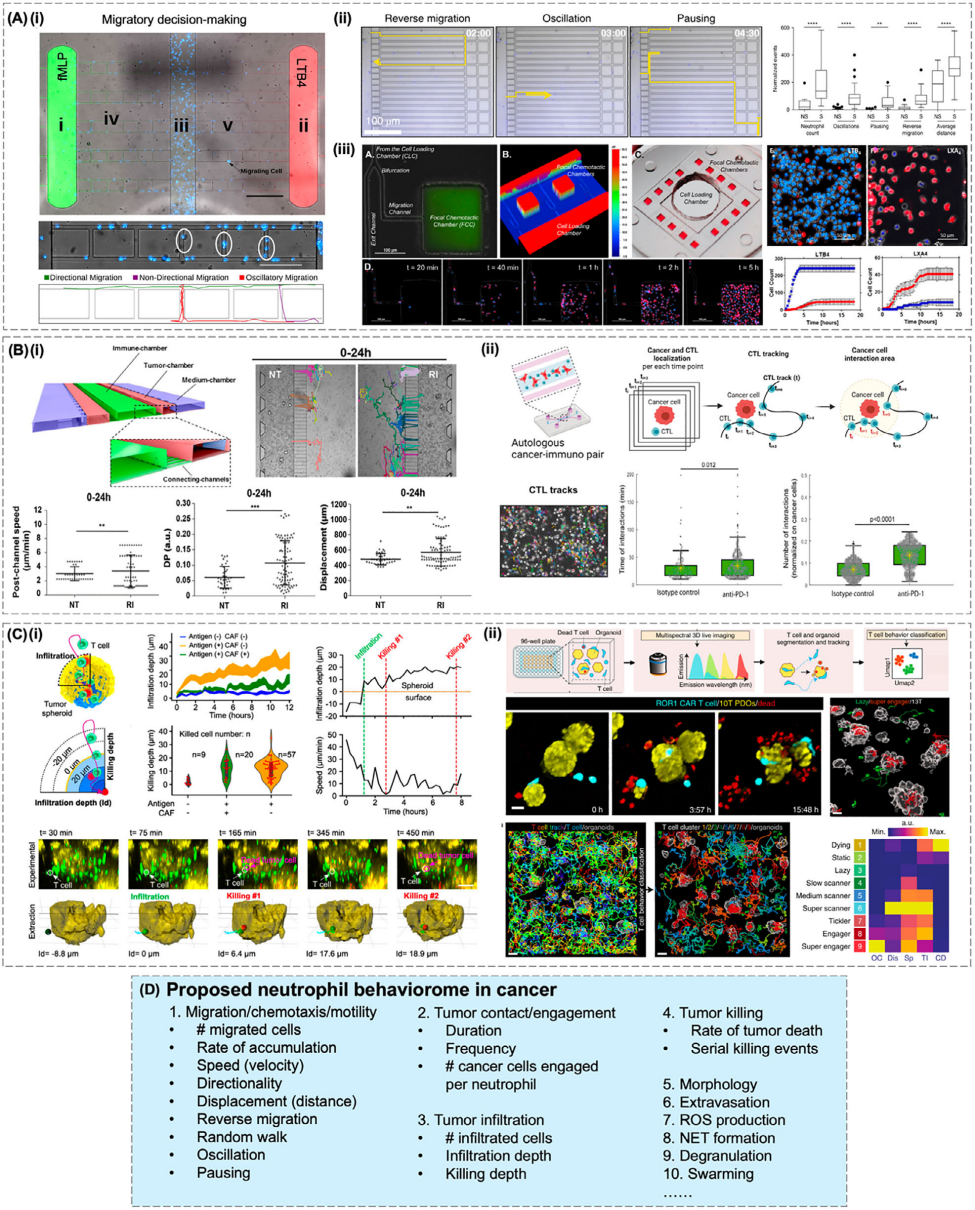
MPS‐based profiling of immune cell behavioromes. (A) Microfluidics‐based profiling of neutrophil behaviorome in health and non‐cancer diseases. (i) A microfluidic chip to quantify migratory decision‐making of human neutrophils in the central chamber in response to two competitive chemoattractants in the side chambers: pro‐resolution fMLP and pro‐inflammatory LTB4. The microchannel mazes connecting the main chambers enable quantification of directional, non‐directional, and oscillatory migration. Adapted with permission from ref. [169], Copyright 2019 Frontiers. (ii) A microfluidic device to measure spontaneous neutrophil motility from a droplet of blood for sepsis diagnosis. Motility parameters including neutrophil count, reverse migration, oscillation, pausing, and average distance were measured to feed into a machine learning algorithm for predicting patient status (sepsis vs. non‐sepsis). Adapted with permission from ref. [245], Copyright 2018 Springer Nature. (iii) A microfluidic model to measure interactions between neutrophils and monocytes during recruitment to inflammation sites. Leucocytes from the cell loading chamber can migrate into the focal chemotactic chambers pre‐loaded with chemoattractants. Neutrophils (blue) and monocytes (red) show distinct migration dynamics to chemoattractants LTB_4_ and LXA_4_ over time. Adapted with permission from ref. [238], Copyright 2012 National Academy of Sciences. (B) Microfluidics‐based profiling of non‐neutrophil immune cell behavioromes in cancer. (i) A 3D microfluidic device to measure dendritic cell migration toward untreated (NT) and chemotherapy‐treated (RI) colorectal cancer cells. Pre‐channel and post‐channel speeds, directional persistence (DP), and displacement were measured. Adapted with permission from ref. [253], Copyright 2017 Springer Nature. (ii) A microfluidic chip to assess lung cancer patient‐specific responses to anti‐PD‐1 therapy using autologous cancer cell‐T cell pairs. Tracking of T cell movement near a cancer cell enables the quantification of both the time duration and the number of T cell‐cancer cell interactions or direct contact. CTL, cytotoxic T lymphocyte. Adapted with permission from ref. [256], Copyright 2024 Elsevier. (C) Spheroid and organoid‐based profiling of T cell behavioromes in cancer. (i) A T cell‐tumor spheroid co‐culture system to track the temporal dynamics of T cell (green) infiltration into and cytotoxicity against tumor spheroids (yellow), including T cell speed, infiltration depth, and killing depth. Adapted with permission from ref. [259], Copyright 2022 National Academy of Sciences. (ii) A T cell‐tumor organoid co‐culture system, combined with 3D live‐cell imaging and computational analysis, to measure dynamic behaviors of engineered T cells and classify them into nine clusters ranging from “lazy” to “super engager”. The T cell behaviors measured include organoid contact (OC), square displacement (Dis), speed (Sp), T cell interactions (TI), and T cell death (CD). PDO, patient‐derived organoid. Adapted with permission from ref. [261], Copyright 2023 Springer Nature. (D) A proposed list of neutrophil behaviors measurable during tumor interactions, reflecting ongoing efforts to profile a comprehensive neutrophil behaviorome in cancer. #, the number of. ROS, reactive oxygen species. NET, neutrophil extracellular trap.

Although intravital microscopy makes live‐cell imaging also possible in animal models, MPS allow long‐term, continuous monitoring for hours to days and quantitative single‐cell tracking of human cells. Microfluidic devices also provide greater optical accessibility, more precise environmental control, and higher experimental throughput than animals [17, 19, 20, 160]. Due to these benefits, MPS have been used to investigate human neutrophil behaviors in a variety of inflammatory diseases beyond cancer, including bacterial [166, 221], fungal [222, 223, 224, 225], and viral [226] infections, sepsis [227, 228], asthma [229], chronic obstructive pulmonary disease (COPD) [229], neurodegeneration [230], cytokine storms [231], and non‐alcoholic fatty liver disease (NAFLD) [232]. Using these complex in vitro models, a wide range of neutrophil behaviors have been measured as functional readouts, including extravasation [166, 226, 229, 233, 234], chemotaxis and migration [169, 172, 235, 236, 237, 238], swarming [224, 237], NET formation [239, 240, 241, 242], ROS production [241, 243], and phagocytosis [166, 244]. After extravasation and entering an infected tissue, neutrophils perform a crucial function called chemotaxis, which is directional migration along a chemoattractant gradient toward the source of inflammation, before they can find and attack the infectious pathogens [22]. Neutrophil chemotaxis, sometimes broadly referred to as migration, has been extensively investigated using MPS platforms by research groups led by Daniel Irimia and Caroline Jones. These teams have developed microfluidic devices with maze‐like architectures and chambers to examine the migratory decision‐making behavior of human neutrophils under both healthy and infectious conditions (Figure 8A). Using time‐lapse images of these devices, the researchers tracked neutrophil trajectories on a single‐cell level and quantified a variety of clinically relevant parameters such as rate of accumulation, speed or velocity, directionality, displacement or distance, reverse migration, oscillation, pausing, and non‐directional migration [169, 172, 222, 228, 235, 236, 238, 245, 246]. Such detailed, complex, and nuanced measurements provide valuable insight into the spatiotemporal dynamics of neutrophil migration that are difficult to quantify otherwise. Many of these behavioral parameters of neutrophils are also highly relevant to cancer and could be quantified in MPS to characterize the behaviorome of neutrophils in cancer in future studies.

Another group of studies has used MPS, including microfluidic systems and tumor organoids, to measure the real‐time behaviors of immune cells other than neutrophils in cancer [247]. Research groups led by Luca Businaro and Maria Carla Parrini have developed microfluidic devices, coupled with live‐cell imaging and computational algorithms for automated image analysis [248, 249], to quantify a wide range of behaviors of both immune cells (T cells, peripheral blood mononuclear cells (PBMCs), and dendritic cells) and cancer cells (Figure 8B). These behaviors include immune cell migration (chemotaxis, motility, speed, displacement, directionality, track curvature, and random walks) [215, 250, 251, 252, 253, 254, 255], kinetics of immune cell‐cancer cell direct interaction (contact duration and frequency) [215, 253, 256, 257], dendritic cell phagocytosis of cancer cells [253], and cancer cell invasion, motility, and death or apoptosis [215, 250, 256, 257]. In some cases, the researchers also evaluated these behavioral changes in response to chemotherapy [252, 254, 255] and multiple classes of immunotherapy, including anti‐PD‐1 immune checkpoint blockade [256], anti‐HER2 ADCC [257], oncolytic vaccinia virus [215], and dendritic cell activation [253]. Choi et al. used a vascularized microfluidic device to quantitatively compare tissue‐resident NK cells and conventional NK cells in terms of migration, tumor infiltration, dynamic interaction with cancer cells, and cytotoxicity using live‐cell imaging and cell tracking analysis [258]. Ao et al. developed an automated high‐throughput microfluidic platform to track the temporal dynamics of T cell infiltration and cytotoxicity within CAF‐incorporated tumor spheroids, including T cell speed, infiltration depth over time, and killing depth (Figure 8Ci). Using this platform, they discovered an epigenetic drug that increased T cell‐tumor infiltration and improved the anti‐tumor efficacy of anti‐PD‐1 therapy [259]. Similarly, Anne C. Rios's group developed BEHAV3D, an innovative system combining 3D high‐resolution live‐cell imaging and computational analysis to dissect the dynamic interactions between engineered T cells and patient‐derived tumor organoids. The system enables tracking and quantitative analysis of T cell migration and motility patterns, frequency and duration of T cell‐tumor contact, number of cancer cells engaged per T cell, tumor killing rate of T cells, and serial killing events [260]. Using BEHAV3D, Dekkers et al. identified distinct behavioral states of therapeutic T cells, including a “super engager” subset capable of serial tumor killing, revealing functional heterogeneity among cellular immunotherapies [261] (Figure 8Cii). These powerful methodologies developed for T cells could also be applied to measuring the behaviorome of neutrophils.

Despite the inherent compatibility of MPS with time‐lapse imaging, current microfluidic organ‐on‐a‐chip studies remain largely confined to end‐point imaging assays such as live/dead and immunofluorescence staining [215]. A recent study by Yuan et al. features a deep learning‐powered cancer‐on‐a‐chip system to quantify phenotypic drug responses of patient‐derived cancer cells using longitudinal brightfield imaging, enabling label‐free, real‐time assessment that is more convenient than conventional fluorescence‐based imaging [262]. Greater adoption of real‐time assays is needed to establish a more comprehensive behaviorome of human neutrophils in cancer, which would provide critical insights into the details of neutrophil‐cancer interactions and modes of action of neutrophil‐based cancer immunotherapies (Figure 8D).

### 7.4 Integrating the Neutrophil Behaviorome With Multi‐Omics

Recent advances in multi‐omics technologies—including genomics, transcriptomics, proteomics, and metabolomics—have transformed cancer immunology by enabling comprehensive, systems‐level characterization of tumor‐immune interactions and the tumor microenvironment. These approaches, particularly when combined with single‐cell and spatial dimensions, have become prominent for dissecting immune heterogeneity and identifying biomarkers that inform immunotherapy response [263, 264, 265, 266]. Extending beyond static and molecular profiling, the behaviorome will add a valuable dimension of dynamic and functional phenotyping to the existing multi‐omics paradigm. Recent MPS‐based studies have integrated behaviorial and functional measurements with targeted molecular assays and multi‐omics approaches to link dynamic immune‐tumor interactions with their underlying molecular programs. For example, Manoharan et al. combined a microfluidic tumor‐on‐a‐chip model with cytokine profiling and flow cytometry to reveal the role of CCL5 and leptin in the intricate interplay between macrophages, T cells, and breast cancer cells [267]. Using the same tumor‐on‐a‐chip device, Ravi et al. performed both functional assays in the device (real‐time migration, invasion, and morphology) and single‐cell RNA sequencing on retrieved cells off the device to uncover the synergistic effect of CAFs and macrophages on breast cancer progression and identify the kynurenine metabolic pathway as a potential immune evasion mechanism [268]. To evaluate CAR‐T cell therapy for glioblastoma, Ravi et al. combined an updated microfluidic device with multiplexed cytokine profiling to measure both real‐time dynamic CAR‐T cell‐cancer interactions and the secretome of CAR‐T cells [269]. Building on T cell‐tumor organoid co‐cultures, Wezenaar et al. developed a behavior‐guided transcriptomics (BGT) protocol that links live‐cell imaging data (T cell motility, tumor engagement, and cytotoxicity metrics) with single‐cell gene expression programs to identify molecular drivers behind T cell functional heterogeneity and new biomarkers that can guide more effective immunotherapy [270]. Likewise, Tostado et al. built a microfluidic cell‐trapping platform to capture paired NK cell‐cancer cell interactions using live‐cell imaging. The authors then combined imaging‐based data with downstream single‐cell transcriptomic analysis to identify molecular mechanisms underlying heterogeneous responses to NK cell cytotoxicity [271]. Using a vascularized lung tumor‐on‐a‐chip system, Campisi et al. combined functional readouts including NK cell extravasation, tumor infiltration, and tumor cytotoxicity with single‐cell RNA sequencing to reveal the important role of vascular Stimulator of Interferon Genes (STING) signaling in the antitumor activity of NK cells [272]. Recently, a vascularized liver tumor‐on‐a‐chip OrganiX platform developed by Vasudevan et al. incorporated spatial transcriptomics and spatial proteomics (multiplexed immunofluorescence imaging) with functional readouts such as CAR‐T cell infiltration, tumor cytotoxicity, and exhaustion to identify pro‐inflammatory factors that could improve CAR‐T cell efficacy [273]. Utilizing the same OrganiX platform, Giustarini et al. integrated functional assays including tumor invasion and angiogenesis with single‐cell RNA sequencing, proteomics, and spatial transcriptomics to recapitulate an immunosuppressive and therapy‐resistant pancreatic tumor microenvironment with clinically relevant gene expression signatures [274]. Together, these studies demonstrate the power of combining MPS‐based behaviorome profiling with multi‐omics and spatial biology technologies to achieve a more holistic understanding of tumor‐immune interactions and therapeutic mechanisms with an unprecedented level of spatiotemporal resolution. In the future, researchers can utilize multi‐omics technologies to uncover the molecular machinery that drives the dynamic behaviors during human neutrophil‐tumor interactions observed in MPS models [206].

### 7.5 MPS‐Driven Personalized Medicine

MPS are emerging as versatile platforms that expand the scope of precision medicine by using patient‐specific biological information to guide clinical decisions, and this can be achieved through either behavior‐based prediction or therapy selection [275]. Behavioral measurements of patient‐derived cells using MPS can be used to predict clinical outcomes. MPS can also be used for drug screening on patient‐derived cells to select the optimal drug candidate for the specific patient. In this section, we discuss both of these emerging applications of MPS for personalized medicine and their potential to advance neutrophil‐based cancer immunotherapy.

#### 7.5.1 Behavior‐Based Prediction of Patient Outcomes

One application of MPS for personalized medicine is to measure the dynamic behavioral features of patient‐derived immune cells or cancer cells and feed these features into a machine learning or artificial intelligence (AI) algorithm to predict clinical outcomes such as disease diagnosis, prognosis, patient stratification, and therapeutic response. Ellett et al. developed a microfluidic device with channels and mazes to measure spontaneous neutrophil motility from a droplet of human blood from critically ill patients for sepsis diagnosis (Figure 8Aii). The researchers measured neutrophil motility parameters including cell count, reverse migration, oscillation, pausing, and average distance to generate a machine learning‐based scoring system that distinguished sepsis patients from non‐sepsis patients with 97% sensitivity and 98% specificity [245]. A set of recent studies from Lance Kam's group has demonstrated the use of microengineered platforms and sparse clinical samples to quantify T cell behaviors as predictive biomarkers for clinically relevant outcomes, particularly in the context of adoptive cellular immunotherapy for chronic lymphocytic leukemia (CLL). Using micropatterned activating surfaces, Lee et al. showed that T cell pattern alignment, motility, and IL‐2 secretion could be integrated through multi‐factor clustering to stratify CLL donors by expansion capacity, which was not possible using conventional clinical markers [219]. Building on this concept, Wang et al. employed PDMS substrates to quantify T cell morphology through multiple spreading parameters, and applied decision tree and random forest machine learning models to distinguish T cells from healthy vs. CLL donors [220]. Extending these findings, Wang et al. developed a superior deep learning model that used images of short‐term T cell spreading on PDMS substrates of varying stiffnesses to classify donor status and predict long‐term, mechanosensitive expansion capacity of CLL T cells [276]. In addition to functional profiling of immune cells, behavioral measurements of patient‐derived cancer cells in MPS have also been mobilized for predicting patient prognosis. Wong et al. combined microfluidics‐based measurements of cancer cell migration, mechanical squeezing, and proliferation into a composite score using logistic regression to classify glioblastoma patients based on progression‐free survival (short‐term vs. long‐term) with an 86% accuracy and predict recurrence time [277]. Likewise, Hua et al. developed a constriction‐based microfluidic device to measure cancer cell deformability, which was used to predict tumor invasiveness and metastatic potential with the help of a deep learning model [278]. Together, these studies highlight the potential of MPS‐enabled behavioral profiling, combined with machine learning, to generate predictive functional biomarkers that can inform clinical diagnosis and prognosis for specific patients. Future integration of MPS‐based profiling of the neutrophil behaviorome with AI may provide a powerful strategy to predict cancer patient outcomes and guide immunotherapy.

#### 7.5.2 Patient‐Specific Drug Screening

Another application of MPS for personalized medicine is to directly evaluate the efficacy of immunotherapy candidates using patient‐derived immune cells and cancer cells and identify optimal therapeutic strategies for specific patients. Recent studies have employed immune cell‐tumor organoid co‐culture models to evaluate diverse immunotherapeutic modalities. For T cell‐based therapies, patient‐derived organoids have been used to assess CAR‐T cell efficacy in lung cancer [279] and to optimize tumor‐infiltrating lymphocyte (TIL) therapy in neuroendocrine cervical cancer [280], while engineered human brain organoids have also been leveraged to investigate CAR‐T cell function and resistance mechanisms in diffuse midline glioma [281]. In parallel, organoid co‐cultures incorporating endogenous or engineered T cells have enabled screening of combination immunotherapies, including immune checkpoint blockade [282] and epigenetic modulators that enhance antigen presentation and T cell cytotoxicity [283]. Beyond T cell‐centric approaches, tumor organoids have also been applied to evaluate other immunotherapies, such as mesothelin‐targeted nanovaccines in pancreatic cancer [284] and NK cell‐based cytotoxicity in ovarian cancer [285]. Notably, these organoid models capture both inter‐patient variability and intratumoral heterogeneity, revealing differential immunotherapy responses across patients and resistant subpopulations within the same tumor.

Compared to well plates, the low‐volume nature of microfluidic devices makes them particularly well suited for personalized drug screening, as they enable efficient utilization of scarce clinical samples of only a few microliters and a few thousand cells by allowing multiple experimental conditions to be assayed in parallel with minimal cell numbers [257]. As a result, recent studies have used patient‐derived microfluidic organ‐on‐a‐chip models to evaluate cancer immunotherapies. For adoptive cell therapies, tumor‐on‐chip platforms have been applied to assess CAR‐T cell function in solid tumors such as lung adenocarcinoma and mesothelioma [286], as well as in leukemia [287]. Organoid‐on‐chip systems incorporating autologous immune cells have also been used to evaluate anti‐PD‐1 immune checkpoint blockade in pancreatic cancer [288] and to quantify immune‐mediated tumor killing and cytokine responses in lung cancer [289]. In addition, a vascularized organoid‐on‐a‐chip model enabled testing of a tumor‐targeted T cell bispecific antibody in colorectal cancer, which promoted immune infiltration and tumor killing [290]. By integrating patient‐derived tumor and immune cells, these microfluidic platforms capture patient‐specific variability in therapeutic response and hold promise as predictive tools for guiding personalized immunotherapy.

Most MPS‐based studies of neutrophil‐cancer interactions to date have used either primary human neutrophils from healthy donors or the HL‐60 human neutrophil‐like cell line, rather than patient‐derived neutrophils. Future work should focus on the use of autologous neutrophils and tumor cells from the same patient to test individualized responses to novel neutrophil‐targeting therapeutic strategies. Moreover, efficient drug screening requires high‐throughput MPS. A central challenge in the field is balancing biological complexity and physiological relevance with experimental throughput and scalability (Figure 2). Companies such as MIMETAS [291], Xellar Biosystems [262], and OrganoBiotech [292] have addressed this challenge by developing commercially available, high‐throughput organ‐on‐a‐chip platforms that enable scalable and automated drug screening. These systems integrate 32 to 128 microfluidic chips within a standard microplate format that shares the same footprint as 96‐ or 384‐well plates, ensuring compatibility with existing laboratory workflows. Such platforms enable immunologists, cancer biologists, and clinicians to perform multi‐patient, multi‐drug, and multi‐condition screening in a human‐relevant microenvironment and may facilitate evaluation and personalization of neutrophil‐based cancer immunotherapies in the future.

### 7.6 Modeling Neutrophil Crosstalk With Other Immune Cells

The tumor microenvironment is highly complex and heterogeneous, comprising diverse cell types including cancer cells, stromal cells such as fibroblasts and endothelial cells, and various immune cell populations such as neutrophils, macrophages, NK cells, and T cells [153]. Therefore, understanding neutrophil‐cancer interactions requires considering the broader network of neutrophil crosstalk with other cellular components of the tumor microenvironment. MPS platforms are well‐suited for this purpose as they enable the investigation of interactions between specific cell types of interest in isolation without being masked or confounded by numerous other cellular and acellular factors in the crowded in vivo environment [17] (Figure 2). Several MPS‐based studies have examined human neutrophil‐NK cell crosstalk in the context of cancer. Babatunde et al. showed that naïve neutrophils inhibited tumor infiltration and cytotoxicity of NK cells in a spheroid tri‐culture model [196], while Shao et al. found that anti‐tumor neutrophils induced greater NK cell recruitment than pro‐tumor neutrophils in a competitive migration setup in a microfluidic device [209]. Other MPS have been used to investigate human neutrophil interactions with other immune cells in infection and inflammatory contexts. Modeling bacterial infection using Pseudomonas aeruginosa, microfluidic infection‐on‐a‐chip platforms have shown that macrophages suppress neutrophil transendothelial migration [293], while monocytic myeloid‐derived suppressor cells (M‐MDSCs) impair neutrophil chemotaxis, extravasation, and migration via IL‐10‐mediated immunosuppression [294]. Babatunde et al. developed a microfluidic system to model focal sites of both sterile and non‐sterile injury using dermal fibroblast spheroids and bacteria *Staphylococcus aureus*. The researchers found that macrophage‐derived extracellular vesicles regulated neutrophil reverse migration and contributed to the resolution of inflammation in wound healing [295]. Together, these studies demonstrate the utility of MPS in dissecting how neutrophils and other immune cell populations modulate each other's function in different diseases in precisely controlled environments. Future studies are needed to investigate neutrophil interactions with a broader range of immune cell types across both the innate and adaptive immune systems, including T cells, to gain a more comprehensive understanding of the complex roles of neutrophils in cancer [216].

T cells remain central mediators of anti‐tumor immunity and primary targets in cancer immunotherapy, as evidenced by the clinical success of immune checkpoint blockade and adoptive T cell therapies [296, 297]. Therefore, it is important to investigate how neutrophils influence T cell functions in MPS models, an area that remains relatively underexplored. Current tumor‐on‐a‐chip studies mostly focus on the tumor site, but adaptive immune responses are initiated upstream in lymph nodes [298]. Lymph nodes are primary sites of adaptive immune activation where antigen‐presenting cells such as dendritic cells instruct T cells to undergo priming, expansion, and differentiation [299]. Increasing evidence from in vivo murine studies indicates that neutrophils can acquire antigen‐presenting properties under certain conditions [71, 81, 86, 87, 92], thus potentially modulating T cell priming in lymph nodes. Lymph node‐on‐a‐chip systems could address a critical gap by modeling the initiation phase of anti‐tumor immunity, where neutrophils may shape T cell responses before T cells reach the tumor site. A few lymph node‐on‐a‐chip systems have emerged in recent years that aim to recapitulate lymph node architecture and immune cell interactions [300, 301]. Shim et al. developed a two‐compartment microfluidic model that demonstrated tumor‐lymph node crosstalk, where indirect co‐culture of murine tumor and lymph node tissue slices led to suppressed T cell activation [302]. Hallfors et al. built a human lymph node‐on‐a‐chip model that enabled real‐time measurement of B cell and T cell motility and drug responses [303]. Morrison et al. designed a more advanced system integrating lymph node stromal components and perfusable lymphatic vasculature to demonstrate that lymphatic endothelial cells enhance immune cell organization, promote B cell enrichment, and regulate cytokine signaling to support immune cell survival and trafficking [304]. Teufel et al. developed a human lymphoid‐tissue‐on‐chip model that recapitulated antigen‐specific antibody responses against influenza vaccines [305]. Together, these studies highlight the ability of MPS to model key immune cell‐cell interactions in the lymph node, and future incorporation of neutrophils into these systems may provide mechanistic insight into neutrophil‐T cell interactions during the initiation of anti‐tumor immunity. Given the numerous in vivo studies showing that CXCR1/2 inhibition to block neutrophil recruitment improves the efficacy of T cell checkpoint blockade in mice [99, 103, 105, 106, 108, 113], human cell‐based organ‐on‐a‐chip models could also be leveraged to evaluate combination immunotherapies targeting neutrophils and T cells simultaneously for synergistic effects.

### 7.7 Assessing Neutrophil‐Based Immunotherapy in Multi‐Organ‐on‐a‐Chip Systems

As pioneers of the organ‐on‐a‐chip technology, Donald Ingber, Dan Huh, and Sangeeta Bhatia emphasized that organ‐on‐a‐chip systems are designed not to recreate entire organs, but to engineer “minimal functional units” that reproduce key tissue‐ and organ‐level functions [19, 166]. Hence, organ‐on‐a‐chip models, just like any other in vitro or in vivo models, ultimately represent a reductionist approach to mimicking human biology. As famously noted by statistician George E. P. Box, all models are inherently imperfect, but they can still provide meaningful insights when appropriately designed [306]. While most organ‐on‐a‐chip models to date focus on one single organ at a time, recent years have witnessed the emergence of models that integrate two or more organ types within a single platform, bringing the biological complexity to the next level. These are referred to as multi‐organ‐on‐a‐chip systems, sometimes also called “body‐on‐a‐chip” or “human‐on‐a‐chip” systems [160]. Some of these systems were used to evaluate both the efficacy and systemic effects of anti‐cancer drugs by integrating tumor tissues with healthy organs [307]. The anti‐EGFR antibody cetuximab was tested in a lung cancer‐skin chip, showing tumor suppression alongside clinically relevant skin toxicity [308], while small‐molecule inhibitor linsitinib was evaluated in a bone tumor‐heart platform that more accurately predicted limited efficacy and reduced cardiotoxicity observed in patients [309]. Chemotherapeutic agents such as doxorubicin induced cardiotoxicity in tumor‐heart two‐organ systems [310, 311] and multi‐organ systems incorporating cardiac tissue [312]. Liver‐coupled systems further revealed metabolism‐dependent responses, including hepatic activation of tamoxifen and differential toxicity of imatinib across liver and heart tissues [313], as well as activation‐dependent cytotoxicity of ifosfamide in glioblastoma [314]. Without tumor compartments, multi‐organ systems incorporating liver, kidney, and bone marrow [315] or liver, heart, skin, and bone [312] enabled prediction of human pharmacokinetics and pharmacodynamics (PK/PD) of cisplatin [315] or doxorubicin [312]. Together, these studies highlight the utility of multi‐organ MPS for comprehensive, system‐level evaluation of anti‐cancer drug efficacy and off‐target toxicity.

One challenge of neutrophil‐based immunotherapy is that neutrophils are the most abundant immune cells in circulation, and systemic targeting of these cells may disrupt their physiological functions throughout the body. For example, inhibition of neutrophil chemokine receptor CXCR2 is intended to limit neutrophil recruitment to tumor sites [11], but it could also impair neutrophil trafficking to sites of infection or tissue injury, thereby compromising host defense and wound healing. A two‐organ‐on‐a‐chip model incorporating both a tumor compartment and a bacterial infection or injury‐mimicking compartment could be used to determine whether CXCR2 inhibition selectively restricts neutrophil migration toward the tumor while preserving neutrophil homing to the compartment of infection or tissue damage. Neutrophil‐activating drugs [83] aim to enhance the anti‐tumor, cytotoxic capacity of neutrophils, but these reprogrammed cells may induce off‐target toxicity in healthy tissues. A multi‐organ‐on‐a‐chip system integrating a tumor target and other healthy organoids of interest, such as liver and heart, can be used to assess neutrophil recruitment and cytotoxicity across different tissue compartments following drug administration. For genetic engineering‐based cell therapies such as CAR‐neutrophils [129], these cells can be perfused through a vascular channel connecting multiple organ compartments to track their biodistribution and accumulation in real time. A breast cancer‐on‐a‐chip platform was developed by Maulana et al. to assess the cytokine release syndrome (CRS) as an adverse effect of CAR‐T cell therapy [316]. A similar approach can be adopted to monitor cytokine release by CAR‐neutrophils via ELISA or built‐in biosensors [307] and examine any tissue damage caused by CRS in a multi‐organ‐a‐chip model. Together, these proposed approaches highlight the future potential of multi‐organ‐on‐a‐chip systems to systematically evaluate both the efficacy and safety of different types of neutrophil‐based immunotherapies in a human‐relevant setting.

## 8 Concluding Remarks

Historically underappreciated in cancer biology, neutrophils are now recognized as multifaceted regulators of tumor progression with both pro‐ and anti‐tumor functions. Rather than acting solely as terminal effector cells, they are increasingly viewed as dynamic orchestrators of the tumor microenvironment that shape the magnitude, amplitude, and trajectory of immune responses. Foundational insights into these roles have been driven largely by in vivo animal models, which have been instrumental in uncovering the complexity of neutrophil biology in cancer. However, translating these findings to human disease remains challenging, highlighting the need for complementary, human‐relevant approaches. Microphysiological systems (MPS) offer a powerful in vitro platform to bridge this gap by enabling controlled, real‐time interrogation of human neutrophil‐cancer interactions in well‐defined microenvironments. By integrating human cells, spatial compartmentalization, and live‐cell imaging, MPS allow detailed characterization of neutrophil behaviors with high spatiotemporal resolution, which is difficult to capture using traditional tools.

Looking ahead, several key directions are likely to further advance the field. MPS offers opportunities to move beyond static and molecular characterization toward dynamic and functional profiling of multi‐parametric neutrophil behaviors, which may reveal previously unrecognized mechanisms of action of therapy. The integration of behavioral profiling with multi‐omics technologies holds promise for linking dynamic neutrophil phenotypes to underlying molecular programs. The use of patient‐derived neutrophils and tumor cells in MPS platforms can reveal inter‐patient and intratumoral heterogeneity and open new avenues for personalized medicine, including AI‐assisted prediction of clinical outcomes and patient‐specific therapy selection. Incorporation of additional immune cell types and multi‐organ systems will enable a more holistic and systems‐level understanding of neutrophil function across both innate and adaptive immunity, as well as evaluation of systemic effects of neutrophil‐targeting drugs.

The future of cancer immunotherapy will rely on the complementary use of in vivo, in vitro, and in silico models. Rather than replacing existing approaches, MPS should be viewed as a critical component of an integrated framework that links mechanistic insight to clinical translation. This perspective is supported by recent regulatory milestones, including the first FDA‐accepted IND application for a cancer immunotherapy based solely on organ‐on‐a‐chip data without animal testing in October 2025, the US Senate passing of the FDA Modernization Act 3.0 in December 2025, and the FDA's draft guidance supporting new approach methodologies (NAMs) for drug development using human‐centric data in March 2026. Together, these developments underscore the growing recognition of MPS as valuable tools within the broader preclinical toolkit and are poised to accelerate the translation of safe and effective neutrophil‐based cancer immunotherapies.

## Author Contributions

**Shuai Shao**: conceptualization, writing – review and editing, writing – original draft. **Markus Isfeld Diehl**: writing – original draft, writing – review and editing. **Shuai Shao**: conceptualization, writing – review and editing, writing – original draft. **Caroline N. Jones**: conceptualization, writing – review and editing, writing – original draft, supervision, funding acquisition.

## Conflicts of Interest

The authors declare no conflicts of interest.

## Data Availability

Data sharing not applicable to this article as no datasets were generated or analysed during the current study.
